# Transdiagnostic development of internalizing psychopathology throughout the life course up to age 45: a World Mental Health Surveys report

**DOI:** 10.1017/S0033291720004031

**Published:** 2022-08

**Authors:** Ymkje Anna de Vries, Ali Al-Hamzawi, Jordi Alonso, Laura Helena Andrade, Corina Benjet, Ronny Bruffaerts, Brendan Bunting, Giovanni de Girolamo, Silvia Florescu, Oye Gureje, Josep Maria Haro, Aimee Karam, Elie G. Karam, Norito Kawakami, Viviane Kovess-Masfety, Sing Lee, Zeina Mneimneh, Fernando Navarro-Mateu, Akin Ojagbemi, José Posada-Villa, Kate Scott, Juan Carlos Stagnaro, Yolanda Torres, Miguel Xavier, Zahari N. Zarkov, Ronald C. Kessler, Peter de Jonge

**Affiliations:** 1 Department of Developmental Psychology, University of Groningen, Groningen, The Netherlands; 2 Interdisciplinary Center Psychopathology and Emotion regulation, University Medical Center Groningen, University of Groningen, Groningen, the Netherlands; 3 College of Medicine, Al-Qadisiya University, Diwaniya governorate, Iraq; 4 Health Services Research Unit, IMIM-Hospital del Mar Medical Research Institute, Barcelona, Spain; CIBER en Epidemiología y Salud Pública (CIBERESP), Spain; Pompeu Fabra University (UPF), Barcelona, Spain; 5 Núcleo de Epidemiologia Psiquiátrica - LIM 23, Instituto de Psiquiatria Hospital das Clinicas da Faculdade de Medicina da Universidade de São Paulo, Brazil; Section of Psychiatric Epidemiology - LIM 23, Institute of Psychiatry, University of São Paulo Medical School, São Paulo, Brazil; 6 Department of Epidemiologic and Psychosocial Research, National Institute of Psychiatry Ramón de la Fuente Muñiz, Mexico City, Mexico; 7 Universitair Psychiatrisch Centrum - Katholieke Universiteit Leuven (UPC-KUL), Campus Gasthuisberg, Leuven, Belgium; 8 School of Psychology, Ulster University, Londonderry, United Kingdom; 9 IRCCS Istituto Centro San Giovanni di Dio Fatebenefratelli, Brescia, Italy; 10 National School of Public Health, Management and Development, Bucharest, Romania; 11 Department of Psychiatry, University College Hospital, Ibadan, Nigeria; 12 Parc Sanitari Sant Joan de Déu, CIBERSAM, Universitat de Barcelona, Sant Boi de Llobregat, Barcelona, Spain; Department of Psychology, College of Education, King Saud University, Riyadh, Saudi Arabia; 13 Institute for Development, Research, Advocacy and Applied Care (IDRAAC), Beirut, Lebanon; 14 Department of Psychiatry and Clinical Psychology, Faculty of Medicine, Balamand University, Beirut, Lebanon; Department of Psychiatry and Clinical Psychology, St George Hospital University Medical Center, Beirut, Lebanon; Institute for Development Research Advocacy and Applied Care, Beirut, Lebanon; 15 Department of Mental Health, School of Public Health, The University of Tokyo, Tokyo, Japan; 16 Ecole des Hautes Etudes en Santé Publique (EHESP), EA 4057, Paris Descartes University, Paris, France; 17 Department of Psychiatry, Chinese University of Hong Kong, Tai Po, Hong Kong; 18 Survey Research Center, Institute for Social Research, University of Michigan, Ann Arbor, Michigan, USA; 19 UDIF-SM, Servicio Murciano de Salud. IMIB-Arrixaca. CIBERESP-Murcia, Región de Murcia, Spain; 20 Department of Psychiatry, University of Ibadan, Nigeria; 21 Colegio Mayor de Cundinamarca University, Faculty of Social Sciences, Bogota, Colombia; 22 Department of Psychological Medicine, University of Otago, Dunedin, Otago, New Zealand; 23 Departamento de Psiquiatría y Salud Mental, Facultad de Medicina, Universidad de Buenos Aires, Argentina; 24 Center for Excellence on Research in Mental Health, CES University, Medellin, Colombia; 25 Lisbon Institute of Global Mental Health and Chronic Diseases Research Center (CEDOC), NOVA Medical School-Faculdade de Ciências Médicas, Universidade Nova de Lisboa, Lisbon, Portugal; 26 National Center of Public Health and Analyses, Directorate Mental Health and Prevention of Addictions, Sofia, Bulgaria; 27 Department of Health Care Policy, Harvard Medical School, Boston, Massachusetts, USA

**Keywords:** Anxiety disorders, depression, internalizing disorders, latent class growth analysis

## Abstract

**Background:**

Depressive and anxiety disorders are highly comorbid, which has been theorized to be due to an underlying internalizing vulnerability. We aimed to identify groups of participants with differing vulnerabilities by examining the course of internalizing psychopathology up to age 45.

**Methods:**

We used data from 24158 participants (aged 45+) in 23 population-based cross-sectional World Mental Health Surveys. Internalizing disorders were assessed with the Composite International Diagnostic Interview (CIDI). We applied latent class growth analysis (LCGA) and investigated the characteristics of identified classes using logistic or linear regression.

**Results:**

The best-fitting LCGA solution identified eight classes: a healthy class (81.9%), three childhood-onset classes with mild (3.7%), moderate (2.0%), or severe (1.1%) internalizing comorbidity, two puberty-onset classes with mild (4.0%) or moderate (1.4%) comorbidity, and two adult-onset classes with mild comorbidity (2.7% and 3.2%). The childhood-onset severe class had particularly unfavorable sociodemographic outcomes compared to the healthy class, with increased risks of being never or previously married (OR = 2.2 and 2.0, *p* < 0.001), not being employed (OR = 3.5, *p* < 0.001), and having a low/low-average income (OR = 2.2, *p* < 0.001). Moderate or severe (*v*. mild) comorbidity was associated with 12-month internalizing disorders (OR = 1.9 and 4.8, *p* < 0.001), disability (B = 1.1–2.3, *p* < 0.001), and suicidal ideation (OR = 4.2, *p* < 0.001 for severe comorbidity only). Adult (*v*. childhood) onset was associated with lower rates of 12-month internalizing disorders (OR = 0.2, *p* < 0.001).

**Conclusions:**

We identified eight transdiagnostic trajectories of internalizing psychopathology. Unfavorable outcomes were concentrated in the 1% of participants with childhood onset and severe comorbidity. Early identification of this group may offer opportunities for preventive interventions.

## Introduction

Depressive and anxiety disorders are responsible for the largest share of the global burden of disease due to mental disorders (Kassebaum et al., 2016), as a consequence of their high prevalence, early age-of-onset (AOO), and chronic/recurrent course (Bruce et al., 2005; Judd et al., 1998; Kessler et al., 2005a; Penninx et al., 2011; Wardenaar, Conradi, & de Jonge, 2014). Moreover, these disorders are often comorbid with one another (Lahey, Zald, Hakes, Krueger, & Rathouz, 2014; Ormel et al., 2015), and comorbidity is associated with worse outcomes than pure disorders (Hendriks, Spijker, Licht, Beekman, & Penninx, 2013; Penninx et al., 2011).

High levels of comorbidity have been theorized to be due to a shared, underlying vulnerability. Factor analyses aimed at identifying an evidence-based structure of psychopathology have found that depressive and anxiety disorders cluster together in an *internalizing* dimension, while substance use disorders and behavioral disorders cluster together in an *externalizing* dimension (Krueger et al., 2018). These dimensions have been found to explain almost all of the comorbidity between specific disorders (Kessler et al., 2011). Intergenerational transmission of internalizing disorders (from parents to children) also appears to be primarily transdiagnostic rather than disorder-specific (Starr, Conway, Hammen, & Brennan, 2014). Thus, while the original concept of comorbidity implies that people with comorbid disorders have multiple, separate disorders, these lines of research suggest instead that internalizing disorders reflect the same underlying psychopathological process, although the specific clinical manifestation varies between persons and across the life course. For instance, major depressive disorder (MDD) is rare in children, but specific phobia and social anxiety disorder commonly show an onset in this age group (Kessler et al., 2005a), and children with these disorders have an increased risk of later developing depression (Lieb et al., 2016; Stein et al., 2001).

Research into transdiagnostic processes also suggests that internalizing disorders share underlying mechanisms, such as neuroticism or negative affectivity (Barlow, Sauer-Zavala, Carl, Bullis, & Ellard, 2014). Furthermore, some processes originally hypothesized to be relevant for a specific disorder, such as anxiety sensitivity for panic disorder and rumination for MDD, appear to be correlated with one another and more broadly involved in multiple internalizing disorders (Aldao, Nolen-Hoeksema, & Schweizer, 2010; Hong & Cheung, 2015; Norton & Paulus, 2017). This has led to the development of transdiagnostic treatments that target shared processes. Transdiagnostic treatments have generally been found to be as effective as disorder-specific treatments (Newby, McKinnon, Kuyken, Gilbody, & Dalgleish, 2015; Pearl & Norton, 2017).

These findings suggests that it could be worthwhile to focus more attention on internalizing disorders as a group, as the specific clinical manifestation may be less important to outcomes of interest, such as disability, than a person's general liability to internalizing disorders. Such a cross-disorder approach was common in early epidemiological studies that conceptualized (common) mental illness as falling along a single continuum of severity, prior to the development of the Diagnostic and Statistical Manual of Mental Disorders, 3rd edition (DSM-III) and a greater focus on discrete categories of mental disorder (Slade et al., 2015). More recently, epidemiological studies have also commonly examined groups of disorders instead of or in addition to specific disorders (e.g. de Graaf, ten Have, van Gool, & van Dorsselaer, 2012; Nock, Hwang, Sampson, & Kessler, 2010; Olfson *et al*., 2017), although such groupings have often been based on *a priori* decisions (e.g. grouping all anxiety disorders separately from mood disorders) rather than empirical evidence.

However, previous research has not yet taken full advantage of the parsimony of this model and the additional opportunities it provides for exciting new analyses that examine the course of internalizing psychopathology throughout life. In this paper, we use latent class growth analysis (LCGA) to identify transdiagnostic trajectories of internalizing psychopathology that are distinct in terms of AOO or level of comorbidity. Subsequently, we investigate the associations between these trajectories and important health outcomes including current psychopathology, disability, suicidality, and treatment to examine the potential of this approach to identify persons at high risk of poor outcomes.

## Methods

### Study sample

Data came from 23 cross-sectional, population-based surveys. Of these surveys, four were administered in low/lower-middle-income countries (Colombia, Iraq, Nigeria, and Peru), six in upper-middle-income countries (Brazil, Bulgaria, Colombia [Medellin], Lebanon, Mexico, and Romania), and 14 in high-income countries (Argentina, Belgium, France, Germany, Italy, Japan, the Netherlands, New Zealand, Northern Ireland, Portugal, Spain, Spain [Murcia], and the USA), as classified by the World Bank (World Bank, 2008). Respondents were adult household residents. Respondent selection was based mainly on multi-stage clustered area probability samples designed to represent the household population of each country/region. Interviews were conducted face-to-face in respondents’ homes. The current analysis focuses on all respondents (*n* = 24.158) who were 45 years or older at the time of the interview and were interviewed about all relevant internalizing disorders. Four surveys imposed an upper age limit on participation (at age 65), while the other surveys had no upper age limit (see online Supplemental Table 1 for further information about individual surveys). Informed consent was obtained according to protocols endorsed by local Institutional Review Boards. Within-country sampling methods are described in detail elsewhere (Heeringa et al., 2008; Pennell et al., 2008).

### Measures

#### Mental disorders

All mental disorders were assessed with the World Health Organization (WHO) Composite International Diagnostic Interview (CIDI), a fully-structured interview administered by trained lay interviewers, which generates diagnoses according to DSM-IV criteria (Kessler & Üstün, 2004). To reduce respondent burden, interviews were administered in two parts. All respondents completed Part I, which assesses core mental disorders. Part II, which assesses other disorders and correlates, was administered to all respondents with a lifetime Part I diagnosis and a probability subsample of respondents without a lifetime Part I diagnosis. The current study uses the Part II sample, which was weighted to adjust for differential sub-sampling, so that weighted prevalence estimates in the Part II sample are identical to those in the Part I sample.

The internalizing disorders included in the present study were specific phobia, social anxiety disorder, generalized anxiety disorder (GAD), panic disorder, agoraphobia, post-traumatic stress disorder (PTSD), and MDD. These diagnoses have shown generally good concordance with clinical diagnoses based on blinded SCID reappraisal (Haro et al., 2006). AOO of each disorder was assessed using special recall probes that yield more plausible AOO distributions than conventional recall questions (Knäuper, Cannell, Schwarz, Bruce, & Kessler, 1999).

#### Other measures

All respondents were asked about sociodemographic variables, including educational level, marital status, employment status, and income. They were also asked how many days they had been completely unable to work due to physical or emotional health reasons in the month before interview (days out of role) and whether they had seriously thought about suicide, made a suicide plan, or attempted suicide, in the 12 months before interview. Finally, respondents were asked whether they had sought treatment for problems with emotions, mental health, or the use of alcohol or drugs in the past 12 months. Treatment was subdivided into four sectors: specialist mental health, general medical, human services, and complementary or alternative medicine.

### Statistical analyses

A person-year dataset for first onset of each disorder was built using retrospectively-reported AOO of each disorder. Although the CIDI assesses at which age symptoms were last present, it does not assess non-symptomatic periods that may have occurred between the AOO and the last age at which symptoms were present. Therefore, we calculated the number of internalizing disorders that had *developed* for each year in a participant's life (up to age 45), without regard for which disorders were actually *active* in each year. We chose to examine the life course up to age 45 to maintain a reasonable sample size while ensuring that most internalizing disorders had already developed (65% of cases of GAD or MDD to 97% of cases of social anxiety disorder in our sample). Because few participants developed more than four disorders by age 45 (0.3%), we used number of disorders as an ordinal variable ranging from 0 to 4+ . We performed LCGA in MPlus 8, with a class-specific intercept, slope, and quadratic slope. The number of classes was decided upon based on the sample-size-adjusted Bayesian Information Criterion (BIC), while avoiding very small classes (<1% of participants).

Subsequent linear and (multinomial) logistic regression analyses to examine sociodemographic variables, disability, suicidality, and treatment were performed in SAS 9.4, using survey procedures to account for the clustering and weighting of the data. Regression was done using participants' posterior probabilities of belonging to each class. However, we also assigned participants to their most likely class to obtain (marginal) percentages and means for each class (e.g. for the percentage of women in each class). Because the posterior probabilities were generally very high (⩾90% for 98.5% of the sample), results using the most likely class were highly similar to results using posterior probabilities. In our main analyses, the ‘healthiest’ class was used as a reference. However, to compare classes with internalizing psychopathology amongst each other, we also performed additional regression analyses using each class's AOO and severity level [defined by the rounded mean number of internalizing disorders developed by age 45 and categorized as mild (1 disorder), moderate (2 disorders), and severe (3 or more disorders)] as simultaneous predictors. Sociodemographic analyses and marginal percentages were adjusted for age, sex and country of origin of the participant; all other analyses were adjusted only for country of origin. We performed additional analyses by country income level (low/middle income *v*. high income) and tested the interaction between class and country income level to investigate possible cross-national variation. We set *α* = 0.005 (two-sided) to reduce the likelihood of false positive results.

## Results

### Latent class growth analyses

We conducted latent class growth analyses with 2–10 classes (see online Supplemental Table 2 for fit indices). The sample-size-adjusted BIC decreased monotonously across this range, indicating better fit with more classes; however, solutions with >8 classes included one or more very small classes (<1%) with similar trajectories as higher-prevalence classes. We therefore selected the 8-class solution as the best solution. Entropy was very high (0.997), indicating good separation of classes.

**Fig. 1.**
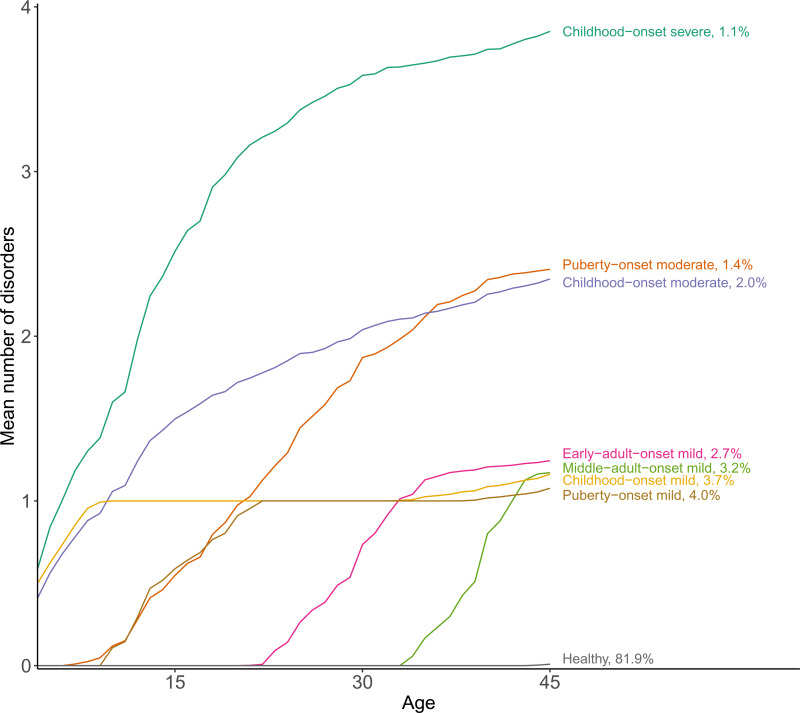
Distinct trajectories of internalizing psychopathology up to age 45. Classes were identified through latent class growth analysis; the trajectories display the actual mean number of disorders by class when respondents are assigned to their most likely class.

Figure 1 shows the mean number of internalizing disorders over time for each class. Most participants (81.9%) were assigned to a healthy class. Three classes showed onset of internalizing psychopathology in childhood and we labelled these the childhood-onset severe (1.1%), childhood-onset moderate (2.0%), and childhood-onset mild (3.7%) classes based on their AOO and the severity level by age 45. Two classes had an onset during puberty, of which one had moderate comorbidity (puberty-onset moderate, 1.4%) and one low comorbidity (puberty-onset mild, 4.0%). Finally, the model identified an early-adult-onset mild class (2.7%), which had onset at around age 30, and a middle-adult-onset mild class (3.2%), which had onset at around age 40.

### Disorder prevalence

**Fig. 2.**
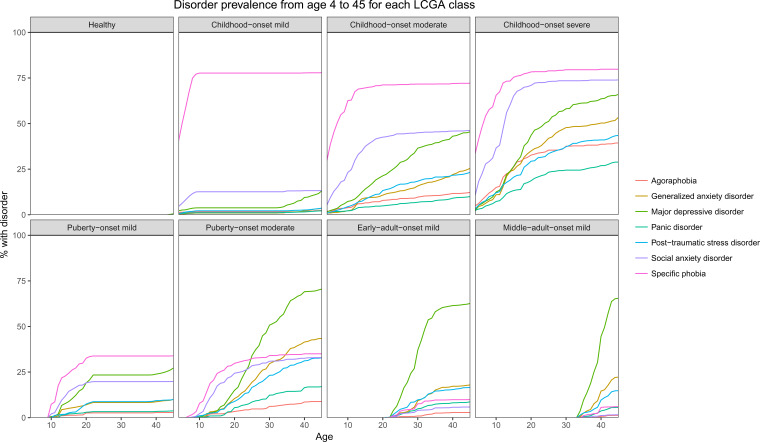
Percentage of participants in each latent class who have developed each individual internalizing disorder from age 4 to 45.

We investigated which disorders were prevalent in each class (Fig. 2). All percentages reported here refer to the percentage of participants who had developed a specific disorder by age 45. Childhood-onset classes were characterized by early specific phobia (reported by 72.1–79.8%). The moderate and severe childhood-onset classes were further characterized by the early onset of social anxiety disorder (reported by 46.2% and 74.0%, respectively), with a later onset of other internalizing disorders, especially MDD (45.5% and 66.0%, respectively). The puberty-onset mild class most frequently developed specific phobia (33.9%), social anxiety disorder (19.8%), or MDD (27.3%) during puberty, with almost no further disorders developing in adulthood. In the puberty-onset moderate class, specific phobia (35.0%) and social anxiety disorder (33.0%) were most frequent in early puberty, but many members of this class later developed MDD (70.5%), GAD (43.4%) or PTSD (32.9%). Finally, the early-adult-onset and middle-adult-onset classes were characterized primarily by MDD (62.6% and 65.5%, respectively).

### Sociodemographic outcomes

Table 1 shows the sociodemographic outcomes within each class (see online Supplemental Table 3 for statistical tests). The healthy class included the lowest percentage of females (51.2%) compared to all other classes (58.8–72.7%, OR = 1.4–2.6, *p* < 0.001). Compared to the healthy class, classes with internalizing psychopathology also tended to be younger (participants aged 65 + : 10.5–22.6% *v*. 31.9%; participants aged 55–64: 26.0–31.8% *v*. 30.2%, OR = 1.4–2.6, *p* < 0.001; participants aged 45–54: 45.6–63.4% *v*. 37.9%, OR = 1.7–5.0, *p* < 0.001).

**Table 1.** Sociodemographic characteristics of each class

Class	*N*	Sex		Age group			Education				Marital status			Employment				Income category			
Female % (SE)		Male % (SE)	45–54 % (SE)	55–64 % (SE)	65 + % (SE)	Low % (SE)	Low-average % (SE)	High-average % (SE)	High % (SE)	Previously married % (SE)	Never married % (SE)	Currently married % (SE)	Home-maker % (SE)	Retired % (SE)	Other % (SE)	Employed % (SE)	Low % (SE)	Low-average % (SE)	High-average % (SE)	High % (SE)
Healthy		16 871	51.2 (0.5)	48.8 (0.5)	37.9 (0.6)	30.2 (0.5)	31.9 (0.6)	30.0 (0.6)	28.8 (0.6)	23.4 (0.6)	17.9 (0.5)	17.4 (0.4)	4.5 (0.2)	78.2 (0.5)	6.0 (0.5)	24.4 (0.8)	11.6 (0.4)	58.0 (0.8)	23.8 (0.5)	24.7 (0.5)	27.9 (0.5)	23.7 (0.5)
Mild	Childhood onset	1 322	67.9 (1.9)	32.1 (1.9)	45.6 (2.1)	31.8 (1.9)	22.6 (1.6)	33.2 (2.1)	30.0 (1.9)	21.7 (1.6)	15.1 (1.4)	22.6 (1.7)	3.2 (0.5)	74.2 (1.8)	6.6 (0.9)	29.1 (2.7)	11.0 (1.4)	53.3 (2.7)	22.7 (1.9)	24.3 (1.8)	29.2 (1.8)	23.8 (1.7)
	Puberty onset	1 528	58.8 (1.9)	41.2 (1.9)	51.6 (1.8)	27.9 (1.5)	20.5 (1.4)	33.3 (2.1)	29.0 (1.6)	22.4 (1.7)	15.4 (1.3)	22.0 (1.5)	5.7 (0.7)	72.4 (1.5)	4.9 (0.7)	23.7 (1.8)	20.8 (2.1)	50.5 (2.5)	25.6 (1.8)	26.0 (1.7)	26.9 (1.6)	21.5 (1.5)
	Early-adult onset	1 152	68.0 (1.9)	32.0 (1.9)	56.4 (2.1)	27.6 (1.8)	16.0 (1.5)	28.4 (2.1)	28.2 (1.9)	25.0 (1.9)	18.4 (1.6)	25.8 (1.9)	5.2 (1.1)	69.0 (2.0)	4.1 (0.7)	26.2 (2.2)	14.8 (1.8)	54.9 (2.5)	26.5 (2.1)	23.7 (1.6)	27.4 (1.8)	22.4 (1.8)
	Middle-adult onset	1 334	59.8 (1.8)	40.2 (1.8)	58.7 (1.8)	28.6 (1.8)	12.7 (1.2)	28.4 (1.9)	29.0 (1.9)	24.0 (1.8)	18.6 (1.5)	25.8 (1.8)	3.1 (0.4)	71.1 (1.9)	5.3 (0.8)	25.6 (2.2)	15.8 (1.8)	53.3 (2.4)	20.7 (1.7)	26.8 (1.7)	30.0 (1.8)	22.5 (1.5)
Moderate	Childhood onset	861	71.3 (2.0)	28.7 (2.0)	57.6 (2.2)	30.2 (2.1)	12.2 (1.4)	30.9 (2.7)	27.9 (2.4)	23.1 (1.9)	18.1 (1.7)	24.0 (1.9)	5.4 (0.8)	70.6 (2.1)	4.6 (0.6)	27.8 (2.6)	21.2 (2.2)	46.5 (2.6)	24.8 (2.0)	24.5 (1.9)	30.7 (2.3)	20.0 (1.7)
	Puberty onset	604	66.4 (2.2)	33.6 (2.2)	56.4 (2.4)	28.6 (2.3)	15.1 (1.6)	30.7 (2.6)	27.1 (2.4)	23.3 (2.3)	18.9 (1.9)	25.2 (2.2)	4.5 (0.8)	70.2 (2.3)	5.2 (0.9)	26.9 (2.7)	17.0 (2.3)	51.0 (3.1)	24.0 (2.3)	26.9 (2.2)	25.3 (2.1)	23.9 (2.2)
Severe	Childhood onset	486	72.7 (2.6)	27.3 (2.6)	63.4 (3.0)	26.0 (2.7)	10.5 (1.7)	39.2 (3.3)	25.7 (2.7)	20.9 (2.3)	14.2 (2.0)	31.0 (2.8)	6.9 (1.1)	62.1 (2.9)	7.9 (1.2)	22.4 (3.5)	28.4 (3.5)	41.3 (4.1)	30.5 (2.7)	31.4 (2.5)	24.4 (2.6)	13.7 (1.5)

Percentages are marginal percentages derived from logistic regression with the sociodemographic characteristic as an outcome and the most likely class as a predictor, controlling for country of origin of respondents and (in the case of education, marital status, employment status, and income category) for age and sex. *N* = unweighted sample size per class (when respondents are assigned to their most likely class).

Unfavorable sociodemographic outcomes were especially common in the childhood-onset severe class, which showed a higher risk than the healthy class of being previously or never married [OR  =  2.2 (95% CI 1.7–2.9) and 2.0 (1.4–2.8), *p* < 0.001], having an ‘other’ employment status (e.g. disabled, unemployed) [OR = 3.5 (2.3–5.1), *p* < 0.001], and having a low or low-average (rather than high) income [OR = 2.2 (1.6–3.1) and 2.2 (1.6–3.0), *p* < 0.001]. All other internalizing classes also had an increased risk of being previously married (OR = 1.4–1.7, *p* < 0.005). The childhood-onset moderate and puberty-onset mild and moderate classes additionally had an increased risk of having an ‘other’ employment status (OR = 1.7–2.3, *p* < 0.005).

Comparing internalizing classes amongst each other, we found significant differences among severity levels in marital status, employment status, and income category. Participants in the severe class had greater risk than those in mild classes of being never or previously married [OR = 2.2 (1.5–3.3) and 1.7 (1.2–2.3), *p* < 0.005], having an ‘other’ employment status [OR = 2.7 (1.7–4.1), *p* < 0.001], and having a low or low-average income [OR = 2.2 (1.5–3.2) and 2.2 (1.5–3.1), *p* < 0.001] (online Supplemental Table 4). Controlling for severity, AOO was not significantly associated with any sociodemographic characteristic.

### Mental health outcomes

Participants in the healthy class rarely reported an active 12-month internalizing disorder (2.4%), but active 12-month disorders were extremely common among childhood-onset classes (69.4%, 79.4%, and 90.6% for mild, moderate, and severe classes, OR = 99.4–474.6, *p* < 0.001) (Table 2). The early-adult-onset and middle-adult-onset mild classes reported much lower rates of 12-month disorders (35.5% and 33.7%, OR = 21.1–23.2, *p* < 0.001), and puberty-onset classes reported intermediate rates (52.0% and 66.5% for the mild and moderate classes, OR = 45.8–88.1, *p* < 0.001). This was confirmed by our analyses using AOO and severity as predictors, which found that puberty or adult onset was associated with reduced risk of a 12-month internalizing disorder [OR = 0.5 (0.4–0.6) and 0.2 (0.2–0.3), *p* < 0.001], while moderate or severe comorbidity was associated with increased risk of a 12-month internalizing disorder [OR = 1.9 (1.6–2.2) and 4.8 (3.1–7.3), *p* < 0.001] (online Supplemental Table 5).

**Table 2.** 12-month active internalizing disorders and days out of role in the past month, by class

Class	*N*	Active 12-month internalizing disorder		Days out of role in the past month	
% (SE)		OR (95% CI)	Mean (SE)	B (SE)
Healthy		16 871	2.4 (0.1)	1.0	1.4 (0.1)	0.0
Mild	Childhood onset	1 322	69.4 (1.8)	99.4 (82.1–120.2)**	1.5 (0.2)	0.1 (0.2)
	Puberty onset	1 528	52.0 (1.8)	45.8 (38.5–54.5)**	2.5 (0.3)	1.2 (0.3)**
	Early-adult onset	1 152	35.5 (1.9)	23.2 (19.1–28.3)**	2.2 (0.2)	0.8 (0.2)**
	Middle-adult onset	1 334	33.7 (1.8)	21.1 (17.5–25.4)**	1.9 (0.3)	0.5 (0.3)
Moderate	Childhood onset	861	79.4 (1.7)	183.0 (143.8–233.0)**	3.0 (0.3)	1.7 (0.3)**
	Puberty onset	604	66.5 (2.3)	88.1 (69.7–111.3)**	3.0 (0.4)	1.7 (0.4)**
Severe	Childhood onset	486	90.6 (1.7)	474.6 (315.8–713.0)**	4.0 (0.4)	2.6 (0.4)**

Percentages are weighted percentages derived from cross-tabulation. Tests were based on logistic regression (for the outcome of having a 12-month active internalizing disorder) or linear regression (for the outcome of days out of role in the past month) using the posterior probabilities of class membership to predict the outcome. All analyses controlled for the country of origin of the participant. * *p* < 0.005 ** *p* < 0.001.

The childhood-onset severe class reported the greatest number of days out of role due to any mental or physical health reason in the past month [mean (SE) = 4.0 (0.4)], which was significantly higher than the mean (SE) of 1.4 (0.1) days out of role reported by healthy participants [B = 2.6 (SE 0.4), *p* < 0.001] (Table 2). The childhood-onset and middle-adult-onset mild classes reported 1.5 (0.2) and 1.9 (0.3) days out of role, which did not differ from the healthy class (*p* = 0.39 and 0.06). The puberty-onset and early-adult-onset mild classes reported 2.2 (0.2) and 2.5 (0.3) days out of role, while the childhood-onset and puberty-onset moderate classes reported 3.0 (0.3) and 3.0 (0.4) (B = 0.8–1.7, *p* < 0.001). Participants in moderate or severe classes reported significantly more days out of role than those in mild classes [B = 1.1 (SE 0.3) and 2.3 (SE 0.5), *p* < 0.001], while AOO was not a significant predictor (online Supplemental Table 6).

With regard to 12-month suicidality (Table 3), all internalizing classes were more likely than the healthy class to report ideation (3.0–13.6% *v*. 0.7%, OR = 4.5–21.7, *p* < 0.001), suicidal plans (0.8–3.5% *v*. 0.1%, OR = 6.6–31.2, *p* < 0.001), or suicide attempts (0.4–1.8% *v*. 0.1%, OR = 5.3–31.9, *p* < 0.005) than the healthy class. The risk of suicidality was strongly associated with level of comorbidity, with the severe class being more likely to report suicidal ideation [OR = 4.2 (2.7–6.6), *p* < 0.001] and both moderate and severe classes being more likely to report suicidal plans [OR = 2.3 (1.3–4.0) and 4.0 (2.0–8.3), *p* < 0.005] than mild classes, although there was no significant association for suicide attempts. AOO was not significantly associated with 12-month suicidality (online Supplemental Table 7).

**Table 3.** 12-month suicidality reported by each class

Class	*N*	Ideation		Plan		Attempt	
% (s.e.)		OR (95% CI)	% (s.e.)	OR (95% CI)	% (s.e.)	OR (95% CI)
Healthy		16 871	0.7 (0.1)	1.0	0.1 (0.0)	1.0	0.1 (0.0)	1.0
Mild	Childhood onset	1 322	3.3 (0.7)	4.5 (2.7–7.4)**	0.9 (0.3)	6.6 (3.5–12.4)**	0.6 (0.2)	7.7 (2.6–23.0)**
	Puberty onset	1 528	3.6 (0.5)	5.1 (3.6–7.4)**	1.0 (0.3)	7.7 (4.1–14.7)**	0.4 (0.2)	5.3 (1.7–16.7)*
	Early-adult onset	1 152	3.4 (0.6)	4.8 (3.2–7.3)**	1.2 (0.3)	9.7 (5.2–18.1)**	0.7 (0.3)	9.8 (3.2–30.3)**
	Middle-adult onset	1 334	3.0 (0.8)	4.3 (2.4–7.6)**	0.8 (0.3)	6.7 (3.3–13.6)**	0.5 (0.2)	7.7 (2.8–20.9)**
Moderate	Childhood onset	861	6.6 (0.9)	9.5 (6.6–13.7)**	2.4 (0.6)	18.5 (9.8–34.6)**	1.0 (0.3)	13.5 (5.1–36.0)**
	Puberty onset	604	4.1 (0.9)	5.7 (3.6–9.3)**	1.7 (0.5)	12.9 (6.9–24.2)**	0.6 (0.2)	8.2 (3.0–22.5)**
Severe	Childhood onset	486	13.6 (1.8)	21.7 (15.1–31.2)**	3.5 (0.9)	31.2 (16.5–59.0)**	1.8 (0.6)	31.9 (13.5–75.7)**

Percentages are weighted percentages derived from cross-tabulation. Tests were based on logistic regression with suicidality as an outcome and the posterior probabilities of class membership as predictors, controlling for country of origin of respondents. *N* = unweighted sample size per class (when respondents are assigned to their most likely class). * *p* < 0.005 ** *p* < 0.001.

All internalizing classes were more likely than the healthy class to report receiving treatment for mental health problems in the past 12 months, in any sector (18.2–49.4% *v*. 5.9%, OR = 3.4–13.4, *p* < 0.001) as well as in each specific sector (online Supplemental Table 8). Receiving treatment was strongly associated with severity, with both moderate and severe classes being more likely to receive any treatment [OR = 2.5 (2.1–3.0) and 3.9 (3.1–4.9) respectively, *p* < 0.001], as well as treatment in specific sectors (online Supplemental Table 9). Receiving any treatment was also associated with AOO, with adult-onset classes having a higher likelihood of receiving any treatment than childhood-onset classes [OR = 1.5 (1.2–1.8), *p* < 0.001], although this association was not significant for any specific treatment sector.

### Low/middle-income *v*. high-income countries

There was a significant difference in prevalence of the different classes between low/middle-income *v*. high-income countries [χ^2^ (7) = 138.7, *p* < 0.001], with the healthy class being more prevalent in low/middle-income countries than in high-income countries (85.5% *v*. 80.0%) and the internalizing classes being less prevalent (online Supplemental Table 10). However, the rank order of prevalence was generally preserved. While there were differences in the prevalence of certain outcomes (e.g. treatment rates), associations between class membership and sociodemographic variables, active 12-month disorders, disability, suicidality, and treatment were generally similar in low/middle-income and high-income countries, with few significant interaction terms (see online Supplemental Tables 11–23).

## Discussion

In this large cross-national survey, we identified transdiagnostic trajectories of internalizing psychopathology by examining the number of internalizing disorders developing up until age 45. Around 18% of our sample developed at least one internalizing disorder and these respondents could be classified into seven classes that showed varying ages of onset and levels of comorbidity. The three childhood-onset classes were characterized by the early onset of specific phobia; moderate and severe childhood-onset classes also showed high rates of early-onset social anxiety disorder and often developed MDD or other internalizing disorders later in life. Adult-onset classes, in contrast, usually only developed MDD, while puberty-onset classes showed a mixed, intermediate pattern.

Classes with internalizing psychopathology were more likely to be female than the healthy class, consistent with a wealth of previous research (Steel et al., 2014). They also tended to be younger. Although this might suggest that younger generations are more likely to develop mental health problems, this finding might also be due to recall bias, with older generations being more likely to forget symptoms that they suffered earlier in life. There were relatively few other sociodemographic differences between the classes, although all internalizing classes were more likely to be previously (rather than currently) married than the healthy class. Surprisingly, educational outcomes did not differ between classes, despite early onset of internalizing disorders in some classes. However, unfavorable sociodemographic outcomes, including a low income and an ‘other’ employment status (e.g. being disabled or unemployed), were clearly concentrated in the childhood-onset severe class.

Compared to the healthy class, all the internalizing classes showed negative mental health outcomes, reporting higher rates of active 12-month internalizing disorders, suicidality, and, for most classes, disability. Internalizing classes also had higher 12-month treatment rates. Furthermore, classes with moderate or severe comorbidity consistently had worse outcomes than classes with mild comorbidity. Controlling for severity, AOO was only associated with the prevalence of active 12-month internalizing disorders and with treatment in any sector. The former may be related to the specific disorders present in childhood-onset *v.* adult-onset classes [e.g. specific phobia, a highly persistent disorder (Wardenaar et al., 2017) *v*. MDD, a more episodic disorder (Bromet et al., 2011)].

We are aware of only two other studies that have traced trajectories of internalizing problems over a larger portion of the lifespan (Colman, Ploubidis, Wadsworth, Jones, & Croudace, 2007; Paksarian et al., 2016), although growth analysis has been used more frequently to analyze the course of internalizing symptomatology in children and adolescents (Edwards et al., 2014; Fanti & Henrich, 2010; Klein et al., 2019; Nivard et al., 2017; Olino, Stepp, Keenan, Loeber, & Hipwell, 2014; Prinzie, van Harten, Dekovic, van den Akker, & Shiner, 2014; Sterba, Prinstein, & Cox, 2007; Weeks et al., 2014). Colman et al. (2007) analyzed a longitudinal dataset (*n* = 4627), in which participants’ current level of depression and anxiety was assessed five times between the ages of 13 and 53 (Colman et al., 2007). The best-fitting model included a large healthy class, an adolescence-limited class, two adolescent-onset persistent classes, and two adult-onset classes. Paksarian et al. (2016) also analyzed a longitudinal dataset (*n* = 591), in which participants were assessed for the past-year presence of psychiatric disorders seven times between the ages of 20 and 50 (Paksarian et al., 2016). The best-fitting model for having any mood or anxiety disorder included a large healthy class; a large class with gradually increasing risk; and a small class in which risk initially increased and subsequently decreased. In contrast to our own work, these studies, based on longitudinal data, could model not just development of symptoms but also recovery. However, neither study covered childhood, and they also did not examine whether particular classes were associated with poor health outcomes. Furthermore, Paksarian et al. (2016) examined the probability of having *any* mood or anxiety disorder, while Colman et al. (2007) examined symptom levels rather than diagnosable disorders. Consequently, neither study is informative with regard to the development of internalizing comorbidity.

Our results show that unfavorable outcomes are highly concentrated in the 1.1% of respondents who reported childhood onset and high levels of comorbidity. Hence, efforts to reduce the burden of mental illness may be most efficient if targeted at this class of people. In our study, this class was characterized by the early co-occurrence of specific phobia and social anxiety disorder, and it would be valuable to further investigate predictors of poor-outcome trajectories. For instance, among children who have already developed specific phobia, can we identify which children will go on to develop other internalizing disorders? In previous research, we found that childhood *generalized* specific phobia (i.e. having multiple phobias) is associated with particularly unfavorable outcomes, even compared to those reporting only a single specific phobia (De Vries et al., 2019), and it is possible that generalized phobia would differentiate between the childhood-onset mild class and the childhood-onset moderate or severe classes. Other predictors, such as childhood adversities, parental socioeconomic status, or family history, might also be highly relevant.

In this study, we did not consider externalizing disorders, due to the limitations of the dataset. Specifically, most countries only assessed behavioral disorders (e.g. attention deficit hyperactivity disorder, oppositional-defiant disorder, and conduct disorder) in young participants (<45 years old); hence, these disorders were not assessed in the included participants. While substance use disorders were assessed in older participants, we felt that these, by themselves, were not fully representative of the externalizing spectrum. However, we know from previous research that comorbidity between internalizing and externalizing disorders is also common (Kessler, Chiu, Demler, & Walters, 2005b), and studying the simultaneous trajectories of internalizing and externalizing psychopathology could therefore also be highly informative. To our knowledge, this work has only been done in children and adolescents; it suggests that internalizing and externalizing trajectories are correlated, although ‘pure’ internalizing and externalizing trajectories are also common (Chen & Simons-Morton, 2009; Fanti & Henrich, 2010; Nivard et al., 2017; Reinke, Eddy, Dishion, & Reid, 2012).

### Strengths and limitations

The main strength of this study is that we used a large cross-national survey, including about 24.000 participants. This gave us enough power to identify and examine the characteristics of small classes. We also investigated a crucial period of life, covering the median ages of onset of all internalizing disorders. Disorders were assessed with a structured interview (CIDI), which has shown moderate-to-good concordance with standardized clinical assessments (Haro et al., 2006), rather than through symptom questionnaires.

The primary limitation of the study is that the data were derived from a cross-sectional interview. While special recall probes were used that yield more plausible distributions of AOO (Knäuper et al., 1999), some recall bias undoubtedly persists. Consistent with this, we found that the healthy class was significantly older than the internalizing classes. It is also possible that people with high symptom levels at the time of interview are more likely to recall symptoms experienced earlier in life, which could potentially lead to spurious associations between the latent trajectory classes and 12-month outcomes. We have previously found that associations between childhood specific phobia and *lifetime* outcomes were present among people with and without psychopathology at the time of interview (De Vries et al., 2019), suggesting that current symptomatology cannot fully explain the association between childhood symptoms and later outcomes. Prospective research has also found consistent associations between childhood-onset disorders and later outcomes (e.g. Lieb et al., 2016); this triangulation [evidence from multiple approaches, each with their own strengths and limitations (Munafò & Davey Smith, 2018)] helps support the plausibility of our findings. However, we cannot fully exclude the possibility that the associations we describe are a consequence of recall bias. The CIDI also provides little information about the longitudinal course of symptoms after onset of a disorder; hence, we could not distinguish between respondents with persistently high levels of comorbidity and respondents that recovered or showed a more episodic course. To confirm and extend our findings, it would be of interest to perform similar analyses in a prospective cohort, although few such cohorts have a sufficiently large sample size and long enough follow-up duration to approximate the analyses we have performed here. Secondly, while we studied the age range in which most internalizing disorders first develop, additional adult-onset trajectories might be discovered if a longer timespan could be studied. We also did not include all disorders that are sometimes considered to be part of the internalizing spectrum (e.g. eating disorders), as these were only assessed in a subset of respondents. Thirdly, it is possible that comorbidity merely reflects a high total symptom count and that a person with, for instance, many symptoms of depression only would have similarly poor outcomes as a person with a few symptoms of several different disorders. However, because the CIDI does not assess all symptoms in participants who do not report criterion symptoms (e.g. depressed mood or loss of interest for MDD), we are unable to calculate a total symptom count to investigate this possibility. Finally, we note that the separation into eight classes should be considered a pragmatic choice rather than a reflection of some real-world mechanism, as we expect that the underlying distributions are continuous rather than discrete. However, the trajectories serve to highlight the importance of AOO and level of comorbidity in predicting unfavorable outcomes.

### Conclusions

In this large, cross-national survey, we identified eight distinct transdiagnostic trajectories of internalizing psychopathology across the lifespan up to age 45. Although all classes with internalizing psychopathology had worse health outcomes than the healthy class, poor outcomes were especially highly concentrated in the 1% of participants with childhood onset and high comorbidity, who had increased risks of unfavorable sociodemographic outcomes as well as high rates of active 12-month disorders, disability, suicidality, and treatment. These findings show the importance of taking a transdiagnostic and life-course-oriented approach to psychopathology, as opposed to a disorder- and episode-specific approach. They also suggest that increased clinical attention to young people likely belong to the high-risk group, for instance children presenting with both specific phobia and social anxiety disorder, may be warranted. Further investigation of the correlates and predictors of membership in this high-risk class may offer opportunities for preventive interventions.

## Supplementary material

For supplementary material accompanying this paper visit https://doi.org/10.1017/S0033291720004031. click here to view supplementary material
