# Supplementary material for: Transdiagnostic development of internalizing psychopathology throughout the life course up to age 45: a World Mental Health Surveys report
Source: Psychol Med. 2020 Nov 10;52(11):2134–43. doi: 10.1017/S0033291720004031 (PMC9367642; doi:10.1017/S0033291720004031)
Supplement: Supplementary file 1 [file S0033291720004031sup001.docx]

|  | **Supplemental table 1. WMH sample characteristics by World Bank income categories*^a^*** | | | | | | | | |
| --- | --- | --- | --- | --- | --- | --- | --- | --- | --- |
|  | |  |  |  |  | **Sample size** | |  |  |
| **Country by income category** | | **Survey*^b^*** | **Sample characteristics*^c^*** | **Field dates** | **Age range** | **Part I** | **Part II** | **Part II & age ≥ 45** | **Response rate*^d^*** |
| **I. Low and lower middle income countries** | | | |  |  |  |  |  |  |
| Colombia | | NSMH | All urban areas of the country (approximately 73% of the total national population). | 2003 | 18-65 | 4,426 | 2,381 | 650 | 87.7 |
| Iraq | | IMHS | Nationally representative. | 2006-7 | 18-96 | 4,332 | 4,332 | 1,105 | 95.2 |
| Nigeria | | NSMHW | 21 of the 36 states in the country, representing 57% of the national population. The surveys were conducted in Yoruba, Igbo, Hausa and Efik languages. | 2002-4 | 18-100 | 6,752 | 2,143 | 751 | 79.3 |
| Peru | | EMSMP | Five urban areas of the country (approximately 38% of the total national population). | 2004-5 | 18-65 | 3,930 | 1,801 | 514 | 90.2 |
| **TOTAL** | |  |  |  |  | (19,440) | (10,657) | (3,020) | 86.5 |
| **II. Upper-middle income countries** | | | |  |  |  |  |  |  |
| Brazil - São Paulo | | São Paulo Megacity | São Paulo metropolitan area. | 2005-8 | 18-93 | 5,037 | 2,942 | 1,118 | 81.3 |
| Bulgaria | | NSHS | Nationally representative. | 2002-6 | 18-98 | 5,318 | 2,233 | 1,492 | 72.0 |
| Colombia – Medellin*^e^* | | MMHHS | Medellin metropolitan area | 2011-12 | 19-65 | 3,261 | 1,673 | 703 | 97.2 |
| Lebanon | | LEBANON | Nationally representative. | 2002-3 | 18-94 | 2,857 | 1,031 | 436 | 70.0 |
| Mexico | | M-NCS | All urban areas of the country (approximately 75% of the total national population). | 2001-2 | 18-65 | 5,782 | 2,362 | 626 | 76.6 |
| Romania | | RMHS | Nationally representative. | 2005-6 | 18-96 | 2,357 | 2,357 | 1,417 | 70.9 |
| **TOTAL** | |  |  |  |  | (24,612) | (12,598) | (5,792) | 77.2 |
| **III. High-income countries** | | | |  |  |  |  |  |  |
| Argentina | | AMHES | Eight largest urban areas of the country (approximately 50% of the total national population) | 2015 | 18-98 | 3,927 | 2,116 | 993 | 77.3 |
| Belgium | | ESEMeD | Nationally representative. The sample was selected from a national register of Belgium residents. | 2001-2 | 18-95 | 2,419 | 1,043 | 557 | 50.6 |
| France | | ESEMeD | Nationally representative. The sample was selected from a national list of households with listed telephone numbers. | 2001-2 | 18-97 | 2,894 | 1,436 | 709 | 45.9 |
| Germany | | ESEMeD | Nationally representative. | 2002-3 | 19-95 | 3,555 | 1,323 | 702 | 57.8 |
| Italy | | ESEMeD | Nationally representative. The sample was selected from municipality resident registries. | 2001-2 | 18-100 | 4,712 | 1,779 | 926 | 71.3 |
| Japan | | WMHJ 2002-2006 | Eleven metropolitan areas. | 2002-6 | 20-98 | 4,129 | 1,682 | 1,135 | 55.1 |
| Netherlands | | ESEMeD | Nationally representative. The sample was selected from municipal postal registries. | 2002-3 | 18-95 | 2,372 | 1,094 | 578 | 56.4 |
| New Zealand*^f^* | | NZMHS | Nationally representative. | 2004-5 | 18-98 | 12,790 | 7,312 | 3,193 | 73.3 |
| N. Ireland | | NISHS | Nationally representative. | 2005-8 | 18-97 | 4,340 | 1,986 | 1,079 | 68.4 |
| Portugal | | NMHS | Nationally representative. | 2008-9 | 18-81 | 3,849 | 2,060 | 990 | 57.3 |
| Spain | | ESEMeD | Nationally representative. | 2001-2 | 18-98 | 5,473 | 2,121 | 1,161 | 78.6 |
| Spain - Murcia | | PEGASUS- Murcia | Murcia region. Regionally representative. | 2010-12 | 18-96 | 2,621 | 1,459 | 828 | 67.4 |
| United States | | NCS-R | Nationally representative. | 2001-3 | 18-99 | 9,282 | 5,692 | 2,495 | 70.9 |
| **TOTAL** | |  |  |  |  | (62,363) | (31,103) | (15,346) | 65.6 |
| **IV. TOTAL** | |  |  |  |  | (106,415) | (54,358) | (24,158) | 71.2 |

*^a^* The World Bank (2012) Data. Accessed May 12, 2012 at: <http://data.worldbank.org/country>. Some of the WMH countries have moved into new income categories since the surveys were conducted. The income groupings above reflect the status of each country at the time of data collection. The current income category of each country is available at the preceding URL.

*^b^* NSMH (The Colombian National Study of Mental Health); IMHS (Iraq Mental Health Survey); NSMHW (The Nigerian Survey of Mental Health and Wellbeing); EMSMP (La Encuesta Mundial de Salud Mental en el Peru); NSHS (Bulgaria National Survey of Health and Stress); MMHHS (Medellín Mental Health Household Study); LEBANON (Lebanese Evaluation of the Burden of Ailments and Needs of the Nation); M-NCS (The Mexico National Comorbidity Survey); RMHS (Romania Mental Health Survey); AMHES (Argentina Mental Health Epidemiologic Survey); ESEMeD (The European Study Of The Epidemiology Of Mental Disorders); WMHJ2002-2006 (World Mental Health Japan Survey); NZMHS (New Zealand Mental Health Survey); NISHS (Northern Ireland Study of Health and Stress);NMHS (Portugal National Mental Health Survey); PEGASUS-Murcia (Psychiatric Enquiry to General Population in Southeast Spain-Murcia);NCS-R (The US National Comorbidity Survey Replication).

*^c^* Most WMH surveys are based on stratified multistage clustered area probability household samples in which samples of areas equivalent to counties or municipalities in the US were selected in the first stage followed by one or more subsequent stages of geographic sampling (e.g., towns within counties, blocks within towns, households within blocks) to arrive at a sample of households, in each of which a listing of household members was created and one or two people were selected from this listing to be interviewed. No substitution was allowed when the originally sampled household resident could not be interviewed. These household samples were selected from Census area data in all countries other than France (where telephone directories were used to select households) and the Netherlands (where postal registries were used to select households). Several WMH surveys (Belgium, Germany, Italy, Spain-Murcia) used municipal, country resident or universal health-care registries to select respondents without listing households. The Japanese sample is the only totally un-clustered sample, with households randomly selected in each of the 11 metropolitan areas and one random respondent selected in each sample household. 14 of the 23 surveys are based on nationally representative household samples.

*^d^* The response rate is calculated as the ratio of the number of households in which an interview was completed to the number of households originally sampled, excluding from the denominator households known not to be eligible either because of being vacant at the time of initial contact or because the residents were unable to speak the designated languages of the survey. The weighted average response rate is 71.2%.

*^e^* Colombia moved from the "lower and lower-middle income" to the "upper-middle income" category between 2003 (when the Colombian National Study of Mental Health was conducted) and 2010 (when the Medellin Mental Health Household Study was conducted), hence Colombia's appearance in both income categories. For more information, please see footnote *a*.

*^f^* For the purposes of cross-national comparisons we limit the sample to those 18+.

Supplemental table 2: Bayesian Information Criterion, entropy, and model-estimated size of smallest class for each latent class growth analysis solution (N=24 158)

| **Number of classes** | **BIC** | **Entropy** | **Model-estimated size of smallest class (%)** |
| --- | --- | --- | --- |
| 2 | 384268.89 | 0.999 | 14.9 |
| 3 | 300261.14 | 0.999 | 3.9 |
| 4 | 251854.68 | 0.998 | 3.7 |
| 5 | 228959.74 | 0.998 | 1.2 |
| 6 | 210461.19 | 0.997 | 1.2 |
| 7 | 199757.94 | 0.998 | 1.1 |
| 8 | 190186.88 | 0.997 | 1.1 |
| 9 | 183223.45 | 0.997 | 0.5 |
| 10 | 177452.32 | 0.997 | 0.5 |

Supplemental table 3: Associations between internalizing classes (posterior probabilities of class membership) and sociodemographic characteristics (outcomes).

| **Class** | | **Sex**  **(ref = male)** | **Age group**  **(ref = 65+)** | | **Education**  **(ref = high)** | | | **Marital status**  **(ref = currently married)** | | **Employment**  **(ref = employed)** | | | **Income category**  **(ref = high)** | | |
| --- | --- | --- | --- | --- | --- | --- | --- | --- | --- | --- | --- | --- | --- | --- | --- |
|  |  | **Female** | **45 - 54** | **55 - 64** | **Low** | **Low-average** | **High-average** | **Previously married** | **Never married** | **Home-maker** | **Retired** | **Other** | **Low** | **Low-average** | **High-average** |
|  | | *OR*  *(95% CI)* | *OR*  *(95% CI)* | *OR*  *(95% CI)* | *OR*  *(95% CI)* | *OR*  *(95% CI)* | *OR*  *(95% CI)* | *OR*  *(95% CI)* | *OR*  *(95% CI)* | *OR*  *(95% CI)* | *OR*  *(95% CI)* | *OR*  *(95% CI)* | *OR*  *(95% CI)* | *OR*  *(95% CI)* | *OR*  *(95% CI)* |
| **Healthy** |  | 1.0 | 1.0 | 1.0 | 1.0 | 1.0 | 1.0 | 1.0 | 1.0 | 1.0 | 1.0 | 1.0 | 1.0 | 1.0 | 1.0 |
| **Mild** | **Childhood onset** | 2.0  (1.7-2.4)** | 1.7 (1.4-2.1)** | 1.5 (1.2-1.8)** | 1.3 (1.0-1.7) | 1.2 (0.9-1.6) | 1.1 (0.8-1.4) | 1.4 (1.1-1.7)* | 0.8 (0.6-1.0) | 1.2 (0.9-1.6) | 1.3 (1.0-1.7) | 1.0 (0.8-1.4) | 0.9 (0.7-1.2) | 1.0 (0.8-1.3) | 1.0 (0.8-1.3) |
|  | **Puberty onset** | 1.4  (1.2-1.6)** | 2.1 (1.7-2.6)** | 1.4 (1.2-1.8)** | 1.3 (1.0-1.7) | 1.2 (0.9-1.5) | 1.1 (0.9-1.4) | 1.4 (1.2-1.6)** | 1.4 (1.1-1.8) | 0.9 (0.7-1.2) | 1.1 (0.9-1.4) | 2.1 (1.6-2.8)** | 1.2 (0.9-1.5) | 1.1 (0.9-1.4) | 1.1 (0.9-1.3) |
|  | **Early-adult onset** | 2.1  (1.7-2.5)** | 3.0 (2.3-3.7)** | 1.8 (1.4-2.3)** | 0.9 (0.7-1.2) | 1.0 (0.7-1.2) | 1.1 (0.8-1.4) | 1.7 (1.4-2.1)** | 1.3 (0.8-2.1) | 0.7 (0.5-1.0) | 1.1 (0.9-1.4) | 1.3 (1.0-1.8) | 1.2 (0.9-1.5) | 1.0 (0.8-1.3) | 1.0 (0.8-1.3) |
|  | **Middle-adult onset** | 1.4  (1.2-1.7)** | 3.9 (3.1-4.9)** | 2.4 (1.9-3.1)** | 0.9 (0.7-1.1) | 1.0 (0.8-1.2) | 1.0 (0.8-1.3) | 1.6 (1.3-2.0)** | 0.8 (0.6-1.0) | 1.0 (0.7-1.3) | 1.1 (0.9-1.4) | 1.5 (1.1-2.0) | 0.9 (0.7-1.2) | 1.1 (0.9-1.4) | 1.1 (0.9-1.4) |
| **Moderate** | **Childhood onset** | 2.4  (2.0-2.9)** | 4.0 (3.0-5.2)** | 2.6 (1.9-3.6)** | 1.0 (0.8-1.4) | 1.0 (0.7-1.3) | 1.0 (0.7-1.3) | 1.5 (1.2-1.9)** | 1.3 (1.0-1.8) | 1.0 (0.7-1.3) | 1.4 (1.1-1.9) | 2.3 (1.7-3.1)** | 1.2 (0.9-1.6) | 1.2 (0.9-1.5) | 1.3 (1.0-1.7) |
|  | **Puberty onset** | 1.9  (1.5-2.3)** | 3.1 (2.4-4.0)** | 2.0 (1.5-2.7)** | 0.9 (0.7-1.3) | 0.9 (0.7-1.2) | 0.9 (0.7-1.3) | 1.6 (1.3-2.0)** | 1.1 (0.8-1.6) | 1.0 (0.7-1.4) | 1.3 (0.9-1.7) | 1.7 (1.2-2.4)* | 1.0 (0.7-1.4) | 1.1 (0.8-1.5) | 0.9 (0.7-1.2) |
| **Severe** | **Childhood onset** | 2.6  (2.0-3.3)** | 5.0 (3.5-7.3)** | 2.6 (1.7-3.9)** | 1.7 (1.1-2.4) | 1.1 (0.8-1.7) | 1.1 (0.8-1.7) | 2.2 (1.7-2.9)** | 2.0 (1.4-2.8)** | 1.8 (1.3-2.6)** | 1.3 (0.8-2.0) | 3.5 (2.3-5.1)** | 2.2 (1.6-3.1)** | 2.2 (1.6-3.0)** | 1.5 (1.1-2.1) |

Table notes: The healthy class is used as the reference class. Estimates are derived from (multinomial) logistic regression with the sociodemographic characteristic as an outcome and the posterior probabilities of class membership as predictors, controlling for country of origin of respondents and, in the case of education, marital status, employment status, and income category, for age and sex. Analyses of education, marital status, employment, and income category controlled for age and sex. * p<0.005 ** p<0.001

Supplemental table 4: Logistic regression analysis of the associations between age of onset and severity of each class (predictors) and sociodemographic characteristics (outcome)

|  |  | **Sex**  **(ref = male)** | **Age group**  **(ref = 65+)** | | **Education**  **(ref = high)** | | | **Marital status**  **(ref = currently married)** | | **Employment status**  **(ref = employed)** | | | **Income category**  **(ref = high)** | | |
| --- | --- | --- | --- | --- | --- | --- | --- | --- | --- | --- | --- | --- | --- | --- | --- |
|  |  | **Female** | **45 – 54** | **55 - 64** | **Low** | **Low-average** | **High-average** | **Previously married** | **Never married** | **Home-maker** | **Retired** | **Other** | **Low** | **Low-average** | **High-average** |
| **Variable** | **Level** | **OR**  **(95% CI)** | **OR**  **(95% CI)** | **OR**  **(95% CI)** | **OR**  **(95% CI)** | **OR**  **(95% CI)** | **OR**  **(95% CI)** | **OR**  **(95% CI)** | **OR**  **(95% CI)** | **OR**  **(95% CI)** | **OR**  **(95% CI)** | **OR**  **(95% CI)** | **OR**  **(95% CI)** | **OR**  **(95% CI)** | **OR**  **(95% CI)** |
| **Age of onset** | Healthy | 0.5  (0.4-0.6)** | 0.6  (0.5-0.7)** | 0.7  (0.5-0.8)** | 0.8  (0.6-1.0) | 0.8  (0.6-1.0) | 0.9  (0.7-1.1) | 0.7  (0.6-0.9)* | 1.1  (0.9-1.4) | 0.9  (0.7-1.1) | 0.8  (0.6-1.0) | 0.8  (0.6-1.0) | 1.0  (0.8-1.3) | 1.0  (0.8-1.2) | 0.9  (0.7-1.1) |
|  | Childhood | 1.0 | 1.0 | 1.0 | 1.0 | 1.0 | 1.0 | 1.0 | 1.0 | 1.0 | 1.0 | 1.0 | 1.0 | 1.0 | 1.0 |
|  | Puberty | 0.7  (0.6-0.8)** | 1.1  (0.9-1.4) | 0.9  (0.7-1.2) | 1.0  (0.8-1.3) | 0.9  (0.7-1.2) | 1.0  (0.8-1.3) | 1.0  (0.8-1.2) | 1.4  (1.0-1.8) | 0.8  (0.6-1.1) | 0.9  (0.7-1.1) | 1.4  (1.0-1.9) | 1.1  (0.8-1.4) | 1.1  (0.9-1.4) | 0.9  (0.7-1.1) |
|  | Adult | 0.8  (0.7-1.0) | 1.9  (1.5-2.4)** | 1.4  (1.1-1.8) | 0.7  (0.5-0.9) | 0.8  (0.6-1.0) | 0.9  (0.7-1.2) | 1.2  (1.0-1.5) | 1.1  (0.8-1.6) | 0.7  (0.5-1.0) | 0.9  (0.7-1.2) | 1.1  (0.8-1.5) | 1.0  (0.8-1.4) | 1.1  (0.8-1.4) | 1.0  (0.8-1.3) |
|  | *Overall test* | *χ^2^ = 14.5, p<0.001* | *χ^2^ = 32.0, p<0.001* | | *χ^2^ = 9.6, p=0.140* | | | *χ^2^ = 8.7, p=0.069* | | *χ^2^ = 13.7, p=0.033* | | | *χ^2^ = 3.9, p=0.686* | | |
| **Severity** | Mild | 1.0 | 1.0 | 1.0 | 1.0 | 1.0 | 1.0 | 1.0 | 1.0 | 1.0 | 1.0 | 1.0 | 1.0 | 1.0 | 1.0 |
|  | Moderate | 1.3  (1.1-1.5) | 1.9  (1.5-2.4)** | 1.6  (1.2-2.1)** | 0.8  (0.6-1.0) | 0.8  (0.6-1.0) | 0.9  (0.7-1.1) | 1.1  (0.9-1.4) | 1.2  (0.9-1.6) | 0.9  (0.7-1.2) | 1.1  (0.9-1.4) | 1.3  (1.0-1.8) | 1.1  (0.8-1.4) | 1.1  (0.8-1.4) | 1.1  (0.8-1.3) |
|  | Severe | 1.3  (1.0-1.7) | 2.8  (1.9-4.2)** | 1.7  (1.1-2.6) | 1.2  (0.8-1.9) | 0.9  (0.6-1.4) | 1.0  (0.7-1.6) | 1.7  (1.2-2.3)* | 2.2  (1.5-3.3)** | 1.6  (1.0-2.4) | 1.0  (0.6-1.6) | 2.7  (1.7-4.1)** | 2.2  (1.5-3.2)** | 2.2  (1.5-3.1)** | 1.4  (0.9-2.0) |
|  | *Overall test* | *χ^2^ = 8.7, p=0.013* | *χ^2^ = 45.6, p<0.001* | | *χ^2^ = 9.0, p=0.172* | | | *χ^2^ = 20.8, p<0.001* | | *χ^2^ = 32.8, p<0.001* | | | *χ^2^ = 27.4, p<0.001* | | |

Table notes: Age of onset and severity were entered into the model simultaneously. Analyses of education, marital status, employment, and income category controlled for age and sex. All analyses controlled for country of origin of the participant. * p < 0.005 ** p < 0.001

Supplemental table 5: Logistic regression analysis of the association between age of onset and severity of each class (predictors) and having an active 12-month internalizing disorder (outcome)

| **Variable** | **Level** | **OR (95% CI)** | **p** |
| --- | --- | --- | --- |
| **Age of onset** | Healthy | 0.0 (0.0-0.0)** | <0.001 |
|  | Childhood | 1.0 | - |
|  | Puberty | 0.5 (0.4-0.6)** | <0.001 |
|  | Adult | 0.2 (0.2-0.3)** | <0.001 |
|  | *Overall test* | *χ^2^ = 247.5* | <0.001 |
| **Severity** | Mild | 1.0 | - |
|  | Moderate | 1.9 (1.6-2.2)** | <0.001 |
|  | Severe | 4.8 (3.1-7.3)** | <0.001 |
|  | *Overall test* | *χ^2^ = 92.7* | <0.001 |

Table notes: Age of onset and severity were entered into the model simultaneously. All analyses controlled for country of origin of the participant. * p < 0.005 ** p < 0.001

Supplemental table 6: Linear regression analysis of the association between age of onset and severity of each class (predictors) and number of days out of role due to any physical or emotional health reason in the past month (outcome)

| **Variable** | **Level** | **B (SE)** | **p** |
| --- | --- | --- | --- |
| **Age of onset** | Healthy | -0.3 (0.2) | 0.065 |
|  | Childhood | 0.0 | - |
|  | Puberty | 0.7 (0.3) | 0.007 |
|  | Adult | 0.3 (0.2) | 0.223 |
|  | *Overall test* | *F = 3.8* | *0.022* |
| **Severity** | Mild | 0.0 | - |
|  | Moderate | 1.1 (0.3)** | <0.001 |
|  | Severe | 2.3 (0.5)** | <0.001 |
|  | *Overall test* | *F = 16.3* | *<0.001* |

Table notes: Age of onset and severity were entered into the model simultaneously. All analyses controlled for country of origin of the participant * p < 0.005 ** p < 0.001

Supplemental table 7: Logistic regression analysis of the association between age of onset and severity of each class (predictors) and 12-month suicidality (outcome)

| **Variable** | **Level** | **Ideation** | | **Plans** | | **Attempts/gestures** | |
| --- | --- | --- | --- | --- | --- | --- | --- |
|  |  | **OR (95% CI)** | **p** | **OR (95% CI)** | **p** | **OR (95% CI)** | **p** |
| **Age of onset** | Healthy | 0.2 (0.1-0.3)** | <0.001 | 0.1 (0.1-0.2)** | <0.001 | 0.1 (0.0-0.3)** | <0.001 |
|  | Childhood | 1.0 |  | 1.0 |  | 1.0 |  |
|  | Puberty | 0.9 (0.6-1.3) | 0.504 | 0.9 (0.5-1.5) | 0.714 | 0.6 (0.3-1.5) | 0.305 |
|  | Adult | 0.9 (0.6-1.4) | 0.598 | 1.1 (0.6-2.0) | 0.858 | 1.1 (0.4-2.8) | 0.874 |
|  | *Overall test* | *χ^2^ = 0.5* | *0.777* | *χ^2^ = 0.2* | *0.890* | *χ^2^ = 1.3* | *0.513* |
| **Severity** | Mild | 1.0 |  | 1.0 |  | 1.0 |  |
|  | Moderate | 1.6 (1.1-2.4) | 0.008 | 2.3 (1.3-4.0)* | 0.003 | 1.7 (0.8-3.9) | 0.190 |
|  | Severe | 4.2 (2.7-6.6)** | <0.001 | 4.0 (2.0-8.3)** | <0.001 | 3.7 (1.3-10.6) | 0.013 |
|  | *Overall test* | *χ^2^ = 39.3* | *<0.001* | *χ^2^ = 17.4* | *<0.001* | *χ^2^ = 6.2* | *0.045* |

Table notes: Age of onset and severity were entered into the model simultaneously. All analyses controlled for country of origin of the participant. * p < 0.005 ** p < 0.001

Supplemental table 8: 12-month treatment rates reported per class

| **Class** | | **N** | **Specialist mental health** | | **General medical care** | | **Human services** | | **CAM** | | **Any treatment** | |
| --- | --- | --- | --- | --- | --- | --- | --- | --- | --- | --- | --- | --- |
|  |  |  | *% (SE)* | *OR (95% CI)* | *% (SE)* | *OR (95% CI)* | *% (SE)* | *OR (95% CI)* | *% (SE)* | *OR (95% CI)* | *% (SE)* | *OR (95% CI)* |
| **Healthy** |  | 16 871 | 2.2 (0.1) | 1.0 | 3.9 (0.2) | 1.0 | 0.4 (0.1) | 1.0 | 0.4 (0.1) | 1.0 | 5.9 (0.2) | 1.0 |
| **Mild** | **Childhood onset** | 1 322 | 7.1 (0.8) | 3.2 (2.4-4.2)** | 12.7 (1.1) | 3.4 (2.8-4.3)** | 2.1 (0.5) | 5.2 (3.2-8.6)** | 1.7 (0.4) | 3.0 (1.7-5.5)** | 18.2 (1.3) | 3.4 (2.8-4.1)** |
|  | **Puberty onset** | 1 528 | 8.3 (0.8) | 3.7 (2.8-4.9)** | 13.3 (1.0) | 3.6 (2.9-4.4)** | 1.4 (0.3) | 3.2 (1.9-5.4)** | 1.8 (0.3) | 3.2 (2.0-5.2)** | 19.7 (1.2) | 3.6 (3.0-4.4)** |
|  | **Early-adult onset** | 1 152 | 11.3 (1.0) | 5.6 (4.4-7.1)** | 17.9 (1.6) | 5.0 (3.9-6.4)** | 2.5 (0.5) | 6.5 (3.9-10.7)** | 1.4 (0.5) | 2.7 (1.4-5.4)* | 26.5 (1.7) | 5.5 (4.5-6.6)** |
|  | **Middle-adult onset** | 1 334 | 9.1 (1.0) | 4.0 (3.1-5.3)** | 16.8 (1.4) | 4.5 (3.6-5.6)** | 2.2 (0.7) | 5.5 (2.6-11.2)** | 2.1 (0.4) | 3.8 (2.3-6.2)** | 24.8 (1.7) | 4.8 (3.9-5.8)** |
| **Moderate** | **Childhood onset** | 861 | 15.0 (1.4) | 7.5 (5.8-9.8)** | 23.9 (1.7) | 7.6 (6.1-9.4)** | 4.4 (1.0) | 10.1 (5.8-17.6)** | 6.0 (1.0) | 10.3 (6.7-15.9)** | 36.3 (1.9) | 8.7 (7.2-10.6)** |
|  | **Puberty onset** | 604 | 14.9 (1.8) | 7.1 (5.1-9.7)** | 26.1 (2.1) | 7.6 (6.0-9.7)** | 3.5 (0.8) | 7.4 (4.3-13.0)** | 4.2 (1.0) | 6.7 (3.8-11.7)** | 38.6 (2.3) | 8.9 (7.1-11.0)** |
| **Severe** | **Childhood onset** | 486 | 22.2 (1.9) | 11.4 (8.7-14.9)** | 34.4 (2.4) | 11.5 (9.1-14.4)** | 6.3 (1.3) | 12.5 (7.5-20.7)** | 7.3 (1.3) | 10.0 (6.6-15.2)** | 49.4 (2.5) | 13.4 (10.9-16.5)** |

Table notes: Percentages are weighted percentages derived from cross-tabulation. Tests were based on logistic regression with treatment as an outcome and the posterior probabilities of class membership as predictors, controlling for country of origin of respondents. N = unweighted sample size per class (when respondents are assigned to their most likely class). * p<0.005 ** p<0.001

Supplemental table 9: Logistic regression analysis of the association between age of onset and severity of each class (predictors) and 12-month treatment (outcome)

| **Variable** | **Level** | **Specialist mental health** | | **General medical care** | | **Human services** | | **CAM** | | **Any treatment** | |
| --- | --- | --- | --- | --- | --- | --- | --- | --- | --- | --- | --- |
|  |  | **OR (95% CI)** | **p** | **OR (95% CI)** | **p** | **OR (95% CI)** | **p** | **OR (95% CI)** | **p** | **OR (95% CI)** | **p** |
| **Age of onset** | Healthy | 0.3 (0.2-0.4)** | <.001 | 0.3 (0.2-0.4)** | <.001 | 0.2 (0.1-0.3)** | <.001 | 0.3 (0.2-0.4)** | <.001 | 0.3 (0.2-0.4)** | <.001 |
|  | Childhood | 1.0 |  | 1.0 |  | 1.0 |  | 1.0 |  | 1.0 |  |
|  | Puberty | 1.1 (0.8-1.3) | 0.628 | 1.0 (0.8-1.2) | 0.781 | 0.7 (0.4-1.1) | 0.123 | 0.8 (0.5-1.2) | 0.278 | 1.0 (0.9-1.2) | 0.614 |
|  | Adult | 1.4 (1.1-1.8) | 0.013 | 1.3 (1.1-1.7) | 0.012 | 1.2 (0.7-2.0) | 0.538 | 0.9 (0.6-1.5) | 0.748 | 1.5 (1.2-1.8)** | <.001 |
|  | *Overall test* | *χ^2^ = 6.8* | *0.034* | *χ^2^ = 7.8* | *0.020* | *χ^2^ = 4.2* | *0.125* | *χ^2^ = 1.2* | *0.545* | *χ^2^ = 17.9* | *<0.001* |
| **Severity** | Mild | 1.0 |  | 1.0 |  | 1.0 |  | 1.0 |  | 1.0 |  |
|  | Moderate | 2.2 (1.7-2.8)** | <.001 | 2.1 (1.7-2.6)** | <.001 | 2.1 (1.3-3.3)* | 0.001 | 2.6 (1.7-4.1)** | <.001 | 2.5 (2.1-3.0)** | <.001 |
|  | Severe | 3.3 (2.4-4.6)** | <.001 | 3.3 (2.6-4.2)** | <.001 | 2.4 (1.4-4.4)* | 0.002 | 2.8 (1.6-4.8)** | <.001 | 3.9 (3.1-4.9)** | <.001 |
|  | *Overall test* | *χ^2^ = 63.2* | *<0.001* | *χ^2^ = 103.0* | *<0.001* | *χ^2^ = 14.9* | *<0.001* | *χ^2^ = 21.5* | *<0.001* | *χ^2^ = 168.9* | *<0.001* |

Table notes: Age of onset and severity were entered into the model simultaneously. All analyses controlled for country of origin of the participant. * p < 0.005 ** p < 0.001

Supplemental table 10: Prevalence of the different LCGA classes in low/middle-income vs. high-income countries

| **Class** | | **Low/middle income countries**  **(total N = 8812)** | | **High income countries**  **(total N = 15346)** | |
| --- | --- | --- | --- | --- | --- |
|  |  | *N* | *% (SE)* | *N* | *% (SE)* |
| **Healthy** |  | 6834 | 85.5 (0.4) | 10037 | 80.0 (0.4) |
| **Mild** | **Childhood onset** | 441 | 3.7 (0.3) | 881 | 3.7 (0.2) |
|  | **Puberty onset** | 440 | 3.4 (0.2) | 1088 | 4.3 (0.2) |
|  | **Early-adult onset** | 305 | 2.1 (0.2) | 847 | 3.0 (0.1) |
|  | **Middle-adult onset** | 354 | 2.2 (0.2) | 980 | 3.6 (0.2) |
| **Moderate** | **Childhood onset** | 227 | 1.5 (0.1) | 634 | 2.3 (0.1) |
|  | **Puberty onset** | 132 | 0.9 (0.1) | 472 | 1.7 (0.1) |
| **Severe** | **Childhood onset** | 79 | 0.6 (0.1) | 407 | 1.4 (0.1) |
|  | | | | | |
| **Test for differences among country income groups** | | *χ^2^(7) = 138.7, p<.001* | | | |

Table notes: Percentages are weighted percentages derived from cross-tabulation. N = unweighted sample size per class (when respondents are assigned to their most likely class).

Supplemental table 11: Sociodemographic characteristics of each class, for low/middle-income and high-income countries separately.

| **Class** | | **N** | **Sex** | | **Age group** | | | **Education** | | | | **Marital status** | | | **Employment** | | | | **Income category** | | | |
| --- | --- | --- | --- | --- | --- | --- | --- | --- | --- | --- | --- | --- | --- | --- | --- | --- | --- | --- | --- | --- | --- | --- |
|  |  |  | Fe-male | Male | 45 - 54 | 55 - 64 | 65+ | Low | Low-ave-rage | High-  ave-rage | High | Previous-ly married | Never married | Currently married | Home-maker | Retired | Other | Employed | Low | Low-ave-rage | High-ave-rage | High |
|  | |  | % (SE) | % (SE) | % (SE) | % (SE) | % (SE) | % (SE) | % (SE) | % (SE) | % (SE) | % (SE) | % (SE) | % (SE) | % (SE) | % (SE) | % (SE) | % (SE) | % (SE) | % (SE) | % (SE) | % (SE) |
| *Low/middle-income countries* | | | | | | | | | | | | | | | | | | | | | | |
| **Healthy** |  | 6834 | 51.5 (0.8) | 48.5 (0.8) | 47.0 (0.9) | 34.4 (0.9) | 18.6 (0.7) | 34.2 (1.1) | 30.4 (1.0) | 20.1 (0.7) | 15.3 (0.9) | 17.3 (0.7) | 3.2 (0.3) | 79.5 (0.7) | 6.3 (0.7) | 21.9 (1.3) | 17.4 (1.0) | 54.3 (1.3) | 30.0 (0.9) | 21.4 (0.7) | 22.1 (0.8) | 26.5 (0.8) |
| **Mild** | **Childhood onset** | 441 | 67.4 (3.4) | 32.6 (3.4) | 53.1 (4.1) | 36.7 (4.1) | 10.3 (1.6) | 37.0 (3.5) | 31.4 (3.2) | 17.7 (2.8) | 14.0 (3.0) | 26.1 (4.1) | 1.8 (0.5) | 72.1 (4.1) | 6.2 (1.4) | 27.2 (4.6) | 18.9 (3.6) | 47.8 (4.6) | 27.1 (3.6) | 21.9 (3.1) | 23.6 (3.5) | 27.5 (3.3) |
|  | **Puberty onset** | 440 | 63.1 (3.4) | 36.9 (3.4) | 52.9 (3.5) | 30.2 (2.8) | 16.9 (3.0) | 41.9 (3.5) | 28.0 (2.9) | 21.6 (2.8) | 8.5 (1.7) | 19.6 (2.7) | 3.4 (0.9) | 77.0 (2.7) | 5.6 (1.2) | 19.9 (2.8) | 35.7 (5.1) | 38.8 (5.0) | 32.8 (3.7) | 23.2 (2.7) | 21.2 (2.5) | 22.8 (3.2) |
|  | **Early-adult onset** | 305 | 68.7 (4.1) | 31.3 (4.1) | 62.8 (4.2) | 28.8 (4.1) | 8.4 (1.8) | 37.9 (4.9) | 27.9 (4.2) | 22.6 (4.1) | 11.7 (2.7) | 24.3 (4.0) | 5.7 (2.6) | 70.1 (4.1) | 4.1 (1.4) | 28.6 (4.9) | 19.9 (4.7) | 47.3 (5.5) | 34.5 (5.2) | 21.3 (3.2) | 23.7 (4.0) | 20.5 (3.6) |
|  | **Middle-adult onset** | 354 | 66.7 (3.4) | 33.3 (3.4) | 55.1 (3.9) | 36.4 (3.7) | 8.5 (2.0) | 33.3 (3.9) | 26.4 (3.0) | 23.9 (3.2) | 16.5 (2.8) | 26.2 (4.5) | 2.4 (0.7) | 71.3 (4.5) | 4.8 (1.1) | 19.4 (3.7) | 18.3 (3.3) | 57.5 (4.5) | 29.6 (3.8) | 20.5 (3.0) | 24.5 (2.9) | 25.4 (3.4) |
| **Moderate** | **Childhood onset** | 227 | 71.9 (4.5) | 28.1 (4.5) | 61.0 (4.7) | 32.3 (4.6) | 6.7 (1.8) | 26.5 (4.4) | 36.5 (4.8) | 19.7 (3.2) | 17.3 (3.6) | 19.9 (3.8) | 2.7 (0.7) | 77.4 (3.9) | 5.1 (1.1) | 25.2 (5.9) | 32.6 (6.2) | 37.1 (5.0) | 27.6 (3.9) | 17.7 (2.9) | 31.5 (5.1) | 23.2 (3.9) |
|  | **Puberty onset** | 132 | 65.5 (5.4) | 34.5 (5.4) | 56.5 (6.1) | 29.4 (6.0) | 14.0 (3.6) | 32.3 (5.2) | 31.7 (5.0) | 19.3 (4.6) | 16.7 (3.9) | 21.7 (4.6) | 1.1 (0.6) | 77.2 (4.6) | 6.6 (1.9) | 15.9 (6.1) | 13.1 (4.6) | 64.4 (7.1) | 26.8 (5.4) | 22.9 (4.4) | 19.8 (4.1) | 30.5 (5.5) |
| **Severe** | **Childhood onset** | 79 | 76.1 (6.5) | 23.9 (6.5) | 61.1 (7.0) | 24.9 (5.8) | 14.0 (5.8) | 34.1 (7.3) | 36.0 (6.6) | 21.0 (4.8) | 9.0 (4.3) | 19.1 (5.8) | 3.1 (1.1) | 77.8 (5.9) | 12.5 (3.6) | 1.1 (0.9) | 31.9 (8.2) | 54.5 (8.4) | 29.9 (6.1) | 28.1 (5.9) | 20.4 (6.5) | 21.6 (5.8) |
| *High-income countries* | | | | | | | | | | | | | | | | | | | | | | |
| **Healthy** |  | 10037 | 51.0 (0.7) | 49.0 (0.7) | 32.0 (0.7) | 26.8 (0.6) | 41.2 (0.8) | 31.0 (0.8) | 28.1 (0.7) | 22.6 (0.8) | 18.2 (0.7) | 17.1 (0.5) | 5.4 (0.3) | 77.5 (0.6) | 5.8 (0.7) | 25.3 (0.9) | 8.8 (0.5) | 60.1 (1.0) | 20.4 (0.6) | 26.1 (0.7) | 31.5 (0.7) | 22.0 (0.7) |
| **Mild** | **Childhood onset** | 881 | 68.2 (2.2) | 31.8 (2.2) | 40.2 (2.4) | 28.0 (1.9) | 31.8 (2.3) | 35.1 (2.7) | 28.8 (2.4) | 21.2 (1.9) | 14.9 (1.5) | 20.3 (1.6) | 4.5 (0.7) | 75.1 (1.7) | 7.0 (1.2) | 29.8 (3.3) | 7.3 (1.0) | 55.9 (3.4) | 20.3 (2.1) | 25.3 (2.2) | 32.7 (2.0) | 21.7 (2.0) |
|  | **Puberty onset** | 1088 | 57.0 (2.2) | 43.0 (2.2) | 48.8 (2.0) | 25.9 (1.7) | 25.2 (1.6) | 32.5 (2.7) | 29.2 (1.9) | 20.6 (1.9) | 17.7 (1.6) | 22.4 (1.7) | 7.3 (0.9) | 70.3 (1.8) | 4.5 (0.9) | 26.1 (2.4) | 13.5 (1.8) | 55.9 (2.9) | 21.8 (1.9) | 27.6 (2.1) | 30.2 (2.1) | 20.3 (1.6) |
|  | **Early-adult onset** | 847 | 67.7 (2.2) | 32.3 (2.2) | 51.6 (2.4) | 26.2 (1.9) | 22.2 (2.2) | 27.0 (2.2) | 28.1 (2.0) | 24.9 (2.0) | 20.0 (1.9) | 26.3 (2.1) | 5.2 (0.9) | 68.5 (2.3) | 3.9 (0.8) | 26.1 (2.5) | 11.8 (1.6) | 58.2 (2.7) | 22.5 (1.9) | 25.0 (2.0) | 30.1 (2.1) | 22.3 (2.0) |
|  | **Middle-adult onset** | 980 | 57.5 (2.1) | 42.5 (2.1) | 57.5 (2.1) | 25.4 (2.0) | 17.1 (1.7) | 28.8 (2.2) | 29.4 (2.3) | 23.1 (2.0) | 18.8 (1.7) | 25.1 (1.8) | 3.6 (0.6) | 71.3 (1.9) | 5.5 (1.1) | 29.2 (2.9) | 12.5 (1.8) | 52.7 (2.7) | 16.7 (1.8) | 29.6 (2.1) | 33.1 (2.1) | 20.5 (1.6) |
| **Moderate** | **Childhood onset** | 634 | 71.0 (2.2) | 29.0 (2.2) | 54.3 (2.5) | 28.6 (2.3) | 17.1 (2.1) | 35.1 (3.3) | 25.3 (2.7) | 22.2 (2.2) | 17.5 (1.9) | 24.9 (2.1) | 7.6 (1.2) | 67.5 (2.4) | 4.1 (0.8) | 29.8 (3.0) | 15.5 (1.8) | 50.7 (3.0) | 22.7 (2.2) | 27.4 (2.4) | 31.8 (2.6) | 18.0 (1.7) |
|  | **Puberty onset** | 472 | 66.5 (2.4) | 33.5 (2.4) | 53.6 (2.6) | 27.4 (2.4) | 19.0 (2.1) | 32.5 (3.1) | 25.3 (2.6) | 22.8 (2.5) | 19.4 (2.1) | 25.9 (2.4) | 6.8 (1.2) | 67.4 (2.5) | 4.7 (1.0) | 31.9 (3.1) | 14.6 (2.1) | 48.8 (3.3) | 21.9 (2.4) | 29.0 (2.6) | 28.1 (2.5) | 21.0 (2.3) |
| **Severe** | **Childhood onset** | 407 | 71.9 (2.8) | 28.1 (2.8) | 61.0 (3.3) | 25.6 (3.0) | 13.4 (2.1) | 42.5 (3.6) | 22.5 (2.8) | 20.2 (2.4) | 14.8 (2.2) | 32.9 (3.0) | 9.4 (1.6) | 57.8 (3.1) | 6.7 (1.3) | 31.5 (4.3) | 20.9 (3.1) | 40.9 (4.5) | 28.4 (2.9) | 33.7 (2.9) | 26.6 (2.9) | 11.3 (1.4) |

Table caption: Percentages are marginal percentages derived from logistic regression with the sociodemographic characteristic as an outcome and the most likely class as a predictor, controlling for country of origin of respondents and (in the case of education, marital status, employment status, and income category) for age and sex. N = unweighted sample size per class (when respondents are assigned to their most likely class).

Supplemental table 12: Associations between internalizing classes (posterior probabilities of class membership) and sociodemographic characteristics (outcomes), and test for interaction with country income group.

| **Class** | | **Sex**  **(ref = male)** | **Age group**  **(ref = 65+)** | | **Education**  **(ref = high)** | | | **Marital status**  **(ref = currently married)** | | **Employment**  **(ref = employed)** | | | **Income category**  **(ref = high)** | | |
| --- | --- | --- | --- | --- | --- | --- | --- | --- | --- | --- | --- | --- | --- | --- | --- |
|  |  | **Female** | **45 - 54** | **55 - 64** | **Low** | **Low-average** | **High-average** | **Previously married** | **Never married** | **Home-maker** | **Retired** | **Other** | **Low** | **Low-average** | **High-average** |
|  | | *OR*  *(95% CI)* | *OR*  *(95% CI)* | *OR*  *(95% CI)* | *OR*  *(95% CI)* | *OR*  *(95% CI)* | *OR*  *(95% CI)* | *OR*  *(95% CI)* | *OR*  *(95% CI)* | *OR*  *(95% CI)* | *OR*  *(95% CI)* | *OR*  *(95% CI)* | *OR*  *(95% CI)* | *OR*  *(95% CI)* | *OR*  *(95% CI)* |
| *Low/middle-income countries* | | | | | | | | | | | | | | | |
| **Healthy** |  | 1.0 | 1.0 | 1.0 | 1.0 | 1.0 | 1.0 | 1.0 | 1.0 | 1.0 | 1.0 | 1.0 | 1.0 | 1.0 | 1.0 |
| **Mild** | **Childhood onset** | 1.9 (1.4-2.6)** | 2.1 (1.4-3.0)** | 1.9 (1.2-3.0)* | 1.2 (0.7-2.3) | 1.2 (0.6-2.1) | 1.0 (0.6-1.9) | 1.7 (1.2-2.6) | 0.6 (0.4-1.1) | 1.1 (0.7-1.8) | 1.6 (0.9-2.7) | 1.2 (0.7-2.0) | 0.9 (0.6-1.4) | 1.0 (0.7-1.6) | 1.0 (0.6-1.6) |
|  | **Puberty onset** | 1.6 (1.2-2.2)* | 1.2 (0.8-1.9) | 1.0 (0.6-1.5) | 2.3 (1.5-3.7)** | 1.7 (1.1-2.7) | 2.1 (1.2-3.4) | 1.2 (0.9-1.7) | 1.1 (0.7-1.9) | 1.2 (0.7-2.1) | 1.2 (0.8-2.1) | 3.0 (1.8-5.1)** | 1.3 (0.8-2.0) | 1.2 (0.8-1.9) | 1.1 (0.7-1.6) |
|  | **Early-adult onset** | 2.1 (1.4-3.1)** | 3.0 (1.9-4.9)** | 1.9 (1.1-3.3) | 1.5 (0.8-2.8) | 1.2 (0.7-2.3) | 1.5 (0.8-2.9) | 1.6 (1.1-2.5) | 2.1 (0.8-5.7) | 0.8 (0.4-1.5) | 1.7 (1.0-3.1) | 1.3 (0.7-2.4) | 1.5 (0.8-2.7) | 1.3 (0.8-2.1) | 1.3 (0.8-2.3) |
|  | **Middle-adult onset** | 1.9 (1.4-2.6)** | 2.6 (1.5-4.3)** | 2.3 (1.3-4.0)* | 0.9 (0.6-1.5) | 0.8 (0.5-1.3) | 1.2 (0.7-1.9) | 1.7 (1.1-2.7) | 0.9 (0.5-1.6) | 0.7 (0.5-1.2) | 0.9 (0.5-1.5) | 1.0 (0.6-1.6) | 1.1 (0.7-1.7) | 1.0 (0.7-1.6) | 1.1 (0.8-1.6) |
| **Moderate** | **Childhood onset** | 2.4 (1.6-3.8)** | 3.7 (2.0-6.9)** | 2.7 (1.4-5.2)* | 0.7 (0.4-1.4) | 1.1 (0.6-2.0) | 0.9 (0.5-1.6) | 1.2 (0.8-2.0) | 1.0 (0.5-1.7) | 1.2 (0.8-1.8) | 1.9 (1.0-3.9) | 2.7 (1.6-4.8)** | 1.1 (0.7-1.8) | 1.0 (0.6-1.6) | 1.6 (0.9-2.8) |
|  | **Puberty onset** | 1.8 (1.1-2.9) | 1.6 (0.9-3.0) | 1.1 (0.6-2.4) | 0.9 (0.5-1.6) | 1.0 (0.5-1.9) | 0.9 (0.4-1.8) | 1.3 (0.8-2.3) | 0.3 (0.1-1.2) | 0.9 (0.5-1.6) | 0.6 (0.2-1.7) | 0.6 (0.3-1.4) | 0.8 (0.4-1.5) | 1.0 (0.5-1.8) | 0.8 (0.4-1.4) |
| **Severe** | **Childhood onset** | 3.0 (1.5-5.9)* | 1.7 (0.6-4.5) | 0.9 (0.3-2.7) | 1.8 (0.6-6.1) | 2.2 (0.7-6.8) | 2.0 (0.7-5.3) | 1.2 (0.6-2.5) | 1.1 (0.5-2.3) | 2.0 (1.0-3.9) | 0.1 (0.0-0.2)** | 1.8 (0.8-4.0) | 1.3 (0.6-2.7) | 1.7 (0.8-3.6) | 1.1 (0.4-2.7) |
| *High-income countries (vs. low/middle-income countries) (interaction effect)* | | | | | | | | | | | | | | | |
| **Healthy** |  | - | - | - | - | - | - | - | - | - | - | - | - | - | - |
| **Mild** | **Childhood onset** | 1.1 (0.7-1.5) | 0.8 (0.5-1.2) | 0.7 (0.4-1.1) | 1.1 (0.6-2.1) | 1.1 (0.5-2.1) | 1.1 (0.6-2.2) | 0.7 (0.4-1.1) | 1.3 (0.7-2.5) | 1.1 (0.6-2.1) | 0.8 (0.4-1.5) | 0.8 (0.4-1.4) | 1.1 (0.7-1.9) | 1.0 (0.6-1.6) | 1.1 (0.6-1.8) |
|  | **Puberty onset** | 0.8 (0.5-1.1) | 2.1 (1.3-3.4)* | 1.7 (1.0-2.7) | 0.5 (0.3-0.8) | 0.6 (0.4-1.1) | 0.4 (0.2-0.8) | 1.2 (0.8-1.8) | 1.3 (0.7-2.4) | 0.7 (0.4-1.2) | 0.9 (0.5-1.5) | 0.6 (0.3-1.1) | 0.9 (0.5-1.5) | 0.9 (0.5-1.5) | 1.0 (0.6-1.5) |
|  | **Early-adult onset** | 1.0 (0.6-1.5) | 1.0 (0.6-1.7) | 0.9 (0.5-1.8) | 0.5 (0.3-1.0) | 0.7 (0.4-1.4) | 0.6 (0.3-1.3) | 1.1 (0.6-1.7) | 0.5 (0.2-1.4) | 0.9 (0.4-1.9) | 0.6 (0.3-1.1) | 1.1 (0.6-2.3) | 0.7 (0.4-1.3) | 0.7 (0.4-1.2) | 0.7 (0.4-1.3) |
|  | **Middle-adult onset** | 0.7 (0.5-1.0) | 1.7 (1.0-3.0) | 1.0 (0.5-1.8) | 1.0 (0.6-1.6) | 1.2 (0.7-2.0) | 0.8 (0.5-1.4) | 0.9 (0.6-1.6) | 0.8 (0.4-1.7) | 1.4 (0.8-2.6) | 1.4 (0.8-2.4) | 1.8 (1.0-3.2) | 0.8 (0.5-1.4) | 1.1 (0.7-1.9) | 1.0 (0.7-1.6) |
| **Moderate** | **Childhood onset** | 1.0 (0.6-1.6) | 1.1 (0.5-2.2) | 1.0 (0.5-2.0) | 1.5 (0.8-3.1) | 0.8 (0.4-1.6) | 1.0 (0.6-1.9) | 1.3 (0.8-2.3) | 1.6 (0.8-3.2) | 0.7 (0.4-1.1) | 0.7 (0.3-1.4) | 0.8 (0.4-1.5) | 1.2 (0.7-2.2) | 1.3 (0.7-2.4) | 0.8 (0.4-1.5) |
|  | **Puberty onset** | 1.1 (0.6-1.8) | 2.2 (1.1-4.4) | 1.9 (0.9-4.2) | 1.1 (0.5-2.2) | 0.8 (0.4-1.7) | 1.0 (0.5-2.2) | 1.3 (0.7-2.3) | 4.1 (1.1-14.9) | 1.1 (0.5-2.3) | 2.4 (0.8-6.8) | 3.4 (1.4-8.1) | 1.4 (0.7-2.8) | 1.2 (0.6-2.3) | 1.2 (0.6-2.5) |
| **Severe** | **Childhood onset** | 0.8 (0.4-1.7) | 3.5 (1.2-9.8) | 3.1 (1.0-9.7) | 0.9 (0.3-3.2) | 0.4 (0.1-1.4) | 0.5 (0.2-1.6) | 2.1 (1.0-4.6) | 2.1 (0.9-5.0) | 0.8 (0.4-1.9) | **33.6 (7.4-152.7)**** | 2.0 (0.8-5.0) | 2.0 (0.9-4.4) | 1.4 (0.6-3.2) | 1.5 (0.6-4.1) |

Table notes: The healthy class is used as the reference class. Estimates are derived from (multinomial) logistic regression with the sociodemographic characteristic as an outcome and the posterior probabilities of class membership as predictors, controlling for country of origin of respondents and, in the case of education, marital status, employment status, and income category, for age and sex. Analyses of education, marital status, employment, and income category controlled for age and sex. Low/middle income countries are taken as the reference category; estimates for high income countries are deviations from the estimate for low/middle income countries (from the interaction effect between posterior probability and country income level). Interaction effects for which the joint test of effects was also significant at p<0.005 are indicated by **bold** font. * p<0.005 ** p<0.001

Supplemental table 13: Logistic regression analysis of the associations between age of onset and severity of each class (predictors) and sociodemographic characteristics (outcome), and test for interaction with country income group

|  |  | **Sex**  **(ref = male)** | **Age group**  **(ref = 65+)** | | **Education**  **(ref = high)** | | | **Marital status**  **(ref = currently married)** | | **Employment status**  **(ref = employed)** | | | **Income category**  **(ref = high)** | | |
| --- | --- | --- | --- | --- | --- | --- | --- | --- | --- | --- | --- | --- | --- | --- | --- |
|  |  | **Female** | **45 – 54** | **55 - 64** | **Low** | **Low-average** | **High-average** | **Previously married** | **Never married** | **Home-maker** | **Retired** | **Other** | **Low** | **Low-average** | **High-average** |
| **Variable** | **Level** | **OR**  **(95% CI)** | **OR**  **(95% CI)** | **OR**  **(95% CI)** | **OR**  **(95% CI)** | **OR**  **(95% CI)** | **OR**  **(95% CI)** | **OR**  **(95% CI)** | **OR**  **(95% CI)** | **OR**  **(95% CI)** | **OR**  **(95% CI)** | **OR**  **(95% CI)** | **OR**  **(95% CI)** | **OR**  **(95% CI)** | **OR**  **(95% CI)** |
| *Low/middle-income countries* | | | | | | | | | | | | | | | |
| **Age of onset** | Healthy | 0.5 (0.4-0.7)** | 0.5 (0.3-0.7)** | 0.5 (0.3-0.8)* | 0.8 (0.4-1.3) | 0.8 (0.5-1.4) | 0.9 (0.5-1.6) | 0.6 (0.4-0.9) | 1.3 (0.8-2.0) | 0.8 (0.5-1.3) | 0.6 (0.4-1.0) | 0.6 (0.4-0.9) | 1.0 (0.7-1.6) | 1.0 (0.6-1.5) | 0.9 (0.6-1.4) |
|  | Childhood | 1.0 | 1.0 | 1.0 | 1.0 | 1.0 | 1.0 | 1.0 | 1.0 | 1.0 | 1.0 | 1.0 | 1.0 | 1.0 | 1.0 |
|  | Puberty | 0.8 (0.6-1.2) | 0.6 (0.3-0.9) | 0.5 (0.3-0.8)* | 1.6 (1.0-2.6) | 1.2 (0.7-2.1) | 1.6 (0.9-3.0) | 0.8 (0.5-1.2) | 1.2 (0.7-2.2) | 0.9 (0.6-1.5) | 0.6 (0.4-1.1) | 1.4 (0.8-2.4) | 1.2 (0.7-2.0) | 1.2 (0.7-1.9) | 0.9 (0.6-1.4) |
|  | Adult | 1.0 (0.7-1.4) | 1.3 (0.8-2.2) | 1.1 (0.6-2.0) | 0.9 (0.5-1.7) | 0.8 (0.4-1.5) | 1.2 (0.6-2.3) | 1.0 (0.6-1.6) | 1.9 (0.9-4.1) | 0.6 (0.3-1.1) | 0.7 (0.4-1.2) | 0.7 (0.4-1.2) | 1.3 (0.8-2.3) | 1.1 (0.7-1.9) | 1.1 (0.7-1.8) |
| **Severity** | Mild | 1.0 | 1.0 | 1.0 | 1.0 | 1.0 | 1.0 | 1.0 | 1.0 | 1.0 | 1.0 | 1.0 | 1.0 | 1.0 | 1.0 |
|  | Moderate | 1.2 (0.8-1.7) | 1.5 (0.9-2.5) | 1.2 (0.7-2.2) | 0.5 (0.3-1.0) | 0.8 (0.4-1.5) | 0.7 (0.4-1.4) | 0.8 (0.5-1.3) | 0.9 (0.5-1.7) | 0.9 (0.6-1.4) | 0.9 (0.4-1.7) | 0.9 (0.5-1.7) | 0.9 (0.6-1.5) | 0.9 (0.5-1.5) | 1.1 (0.7-2.0) |
|  | Severe | 1.5 (0.7-3.1) | 0.8 (0.3-2.3) | 0.5 (0.2-1.5) | 1.4 (0.4-5.1) | 1.8 (0.5-5.6) | 1.7 (0.7-4.5) | 0.7 (0.3-1.5) | 1.4 (0.6-3.0) | 1.7 (0.7-3.7) | 0.0 (0.0-0.1)** | 1.1 (0.5-2.8) | 1.3 (0.6-3.1) | 1.6 (0.7-3.9) | 1.0 (0.3-2.8) |
| *High-income countries (vs. low/middle-income countries) (interaction effect)* | | | | | | | | | | | | | | | |
| **Age of onset** | Healthy | 1.0 (0.7-1.4) | 1.2 (0.8-1.8) | 1.4 (0.9-2.3) | 1.0 (0.5-1.8) | 1.0 (0.6-1.9) | 1.0 (0.6-1.9) | 1.3 (0.9-2.0) | 0.8 (0.5-1.3) | 1.0 (0.6-1.8) | 1.5 (0.8-2.6) | 1.5 (0.9-2.4) | 0.9 (0.5-1.5) | 1.0 (0.6-1.7) | 1.0 (0.6-1.6) |
|  | Childhood | 1.0 | 1.0 | 1.0 | 1.0 | 1.0 | 1.0 | 1.0 | 1.0 | 1.0 | 1.0 | 1.0 | 1.0 | 1.0 | 1.0 |
|  | Puberty | 0.8 (0.5-1.3) | 2.3 (1.3-4.0)* | 2.2 (1.3-3.9)* | 0.5 (0.3-0.9) | 0.7 (0.4-1.3) | 0.5 (0.3-1.0) | 1.5 (0.9-2.4) | 1.1 (0.6-2.1) | 0.8 (0.5-1.5) | 1.5 (0.8-2.8) | 1.0 (0.5-1.9) | 0.8 (0.5-1.5) | 0.9 (0.5-1.6) | 1.0 (0.6-1.7) |
|  | Adult | 0.8 (0.5-1.2) | 1.6 (0.9-2.9) | 1.4 (0.7-2.7) | 0.7 (0.3-1.4) | 1.0 (0.5-2.0) | 0.7 (0.4-1.5) | 1.3 (0.8-2.3) | 0.5 (0.2-1.1) | 1.2 (0.6-2.4) | 1.4 (0.7-2.6) | 2.1 (1.1-4.0) | 0.7 (0.4-1.3) | 0.9 (0.5-1.7) | 0.9 (0.5-1.5) |
| **Severity** | Mild | 1.0 | 1.0 | 1.0 | 1.0 | 1.0 | 1.0 | 1.0 | 1.0 | 1.0 | 1.0 | 1.0 | 1.0 | 1.0 | 1.0 |
|  | Moderate | 1.1 (0.7-1.7) | 1.3 (0.7-2.3) | 1.4 (0.7-2.6) | 1.7 (0.8-3.4) | 0.9 (0.5-1.8) | 1.3 (0.6-2.7) | 1.5 (0.9-2.5) | 1.4 (0.7-2.8) | 0.9 (0.5-1.7) | 1.4 (0.7-2.9) | 1.8 (0.9-3.5) | 1.2 (0.7-2.2) | 1.3 (0.7-2.3) | 0.9 (0.5-1.7) |
|  | Severe | 0.8 (0.4-1.8) | 4.1 (1.3-12.6) | 4.3 (1.3-14.7) | 0.9 (0.2-3.6) | 0.5 (0.1-1.6) | 0.6 (0.2-1.6) | 2.9 (1.3-6.5) | 1.7 (0.7-4.2) | 0.9 (0.3-2.2) | **62.6 (10.5-371.8)**** | 3.0 (1.1-8.2) | 1.9 (0.7-4.8) | 1.5 (0.6-3.8) | 1.5 (0.5-4.6) |

Table notes: Age of onset and severity were entered into the model simultaneously. Analyses of education, marital status, employment, and income category controlled for age and sex. All analyses controlled for country of origin of the participant. Low/middle income countries are taken as the reference category; estimates for high income countries are deviations from the estimate for low/middle income countries (from the interaction effect between posterior probability and country income level). Interaction effects for which the joint test of effects was also significant at p<0.005 are indicated by **bold** font. * p < 0.005 ** p < 0.001

Supplemental table 14: Associations between internalizing classes (posterior probabilities of class membership) and active 12-month disorders (outcome), by country income group.

| **Class** | | **Low/middle income countries**  **(reference group)** | | **High income countries**  **(vs. low/middle)** | | **Test for interaction between income group and posterior probabilities** | |
| --- | --- | --- | --- | --- | --- | --- | --- |
|  |  | *% (SE)* | *OR (95% CI)* | *% (SE)* | *OR (95% CI)* | *F(1,1157)* | *P-value* |
| **Healthy** |  | 2.5 (0.2) | 1.0 | 2.4 (0.1) | - | - | - |
| **Mild** | **Childhood onset** | 78.6 (3.2) | 152.3 (100.2-231.6)** | 64.6 (2.1) | 0.5 (0.3-0.8) | 7.2 | 0.007 |
|  | **Puberty onset** | 58.6 (3.5) | 52.4 (37.5-73.2)** | 49.3 (2.1) | 0.8 (0.5-1.2) | 1.4 | 0.235 |
|  | **Early-adult onset** | 41.0 (4.4) | 27.0 (18.0-40.5)** | 33.6 (2.1) | 0.8 (0.5-1.2) | 1.2 | 0.281 |
|  | **Middle-adult onset** | 44.3 (3.6) | 29.6 (21.4-41.0)** | 30.2 (2.0) | 0.6 (0.4-0.9) | 6.6 | 0.011 |
| **Moderate** | **Childhood onset** | 84.7 (3.4) | 232.4 (129.5-417.1)** | 77.5 (2.0) | 0.7 (0.4-1.3) | 1.3 | 0.256 |
|  | **Puberty onset** | 82.5 (3.6) | 178.1 (103.9-305.3)** | 62.0 (2.8) | 0.4 (0.2-0.7)* | 9.2* | 0.002 |
| **Severe** | **Childhood onset** | 97.9 (2.1) | 2012.9 (254.6-15912.8)** | 89.1 (1.9) | 0.2 (0.0-1.6) | 2.4 | 0.121 |

Table notes: Percentages are weighted percentages derived from cross-tabulation. Tests were based on logistic regression with 12-month internalizing disorder as an outcome and the posterior probabilities of class membership as predictors, controlling for country of origin of respondents. Participants in low/middle income countries are taken as the reference category; estimates for high income countries are deviations from the estimate for low/middle income countries. The interaction term indicates the significance of the interaction between each probability and country income group.

N = unweighted sample size per class (when respondents are assigned to their most likely class). * p<0.005 ** p<0.001

Supplemental table 15: Logistic regression analysis of the association between age of onset and severity of each class (predictors) and active 12-month disorders (outcome), by country income group

| **Variable** | **Level** | **Low/middle income countries**  **(reference group)** | **High income countries**  **(vs. low/middle)** | **Test for interaction between income group and posterior probabilities** | |
| --- | --- | --- | --- | --- | --- |
|  |  | **OR (95% CI)** | **OR (95% CI)** | **F(2,1156)** | **P-value** |
| **Age of onset** | Healthy | 0.0 (0.0-0.0)** | 1.7 (1.1-2.6) | 0.3 | 0.777 |
|  | Childhood | 1.0 | 1.0 |  |  |
|  | Puberty | 0.4 (0.3-0.7)** | 1.2 (0.7-1.9) |  |  |
|  | Adult | 0.2 (0.1-0.3)** | 1.1 (0.7-1.8) |  |  |
| **Severity** | Mild | 1.0 | 1.0 | 0.8 | 0.443 |
|  | Moderate | 2.2 (1.4-3.5)** | 0.8 (0.5-1.4) |  |  |
|  | Severe | 14.3 (1.9-109.8) | 0.3 (0.0-2.6) |  |  |

Table notes: Age of onset and severity were entered into the model simultaneously. All analyses controlled for country of origin of the participant. Participants in low/middle income countries are taken as the reference category; estimates for high income countries are deviations from the estimate for low/middle income countries. The interaction term indicates the significance of the interaction between age of onset/severity and country income group.

* p < 0.005 ** p < 0.001

Supplemental table 16: Associations between internalizing classes (posterior probabilities of class membership) and 12-month days out of role (outcome), by country income group.

| **Class** | | **Low/middle income countries**  **(reference group)** | | **High income countries**  **(vs. low/middle)** | | **Test for interaction between income group and posterior probabilities** | |
| --- | --- | --- | --- | --- | --- | --- | --- |
|  |  | *Mean (SE)* | *B (SE)* | *Mean (SE)* | *B (SE)* | *F(1,1163)* | *P-value* |
| **Healthy** |  | 1.7 (0.1) | 0.0 | 1.2 (0.1) | - | - | - |
| **Mild** | **Childhood onset** | 1.5 (0.2) | 0.0 (0.3) | 1.5 (0.2) | 0.2 (0.4) | 0.2 | 0.644 |
|  | **Puberty onset** | 3.1 (0.6) | 1.6 (0.6) | 2.3 (0.3) | -0.6 (0.7) | 0.7 | 0.401 |
|  | **Early-adult onset** | 2.3 (0.5) | 0.6 (0.5) | 2.1 (0.2) | 0.2 (0.6) | 0.1 | 0.713 |
|  | **Middle-adult onset** | 2.5 (0.4) | 0.8 (0.4) | 1.7 (0.3) | -0.4 (0.5) | 0.6 | 0.435 |
| **Moderate** | **Childhood onset** | 2.6 (0.7) | 1.3 (0.7) | 3.1 (0.4) | 0.5 (0.8) | 0.4 | 0.506 |
|  | **Puberty onset** | 3.4 (0.7) | 2.0 (0.7) | 2.9 (0.4) | -0.5 (0.9) | 0.3 | 0.569 |
| **Severe** | **Childhood onset** | 3.4 (0.8) | 2.1 (0.8) | 4.1 (0.5) | 0.6 (1.0) | 0.4 | 0.532 |

Table notes: Means are weighted means. Tests were based on linear regression with 12-month disability as an outcome and the posterior probabilities of class membership as predictors, controlling for country of origin of respondents. Participants in low/middle income countries are taken as the reference category; estimates for high income countries are deviations from the estimate for low/middle income countries. The interaction term indicates the significance of the interaction between each probability and country income group.

N = unweighted sample size per class (when respondents are assigned to their most likely class). * p<0.005 ** p<0.001

Supplemental table 17: Linear regression analysis of the association between age of onset and severity of each class (predictors) and 12-month days out of role (outcome), by country income group

| **Variable** | **Level** | **Low/middle income countries**  **(reference group)** | **High income countries**  **(vs. low/middle)** | **Test for interaction between income group and posterior probabilities** | |
| --- | --- | --- | --- | --- | --- |
|  |  | **B (SE)** | **B (SE)** | **F(2,1163)** | **P-value** |
| **Age of onset** | Healthy | -0.2 (0.3) | -0.2 (0.4) | 1.1 | 0.317 |
|  | Childhood | 0.0 (0.0) | 0.0 (0.0) |  |  |
|  | Puberty | 1.3 (0.5) | -0.8 (0.6) |  |  |
|  | Adult | 0.6 (0.4) | -0.4 (0.5) |  |  |
| **Severity** | Mild | 0.0 (0.0) | 0.0 (0.0) | 0.1 | 0.895 |
|  | Moderate | 1.0 (0.6) | 0.2 (0.7) |  |  |
|  | Severe | 1.9 (0.8) | 0.4 (1.0) |  |  |

Table notes: Age of onset and severity were entered into the model simultaneously. All analyses controlled for country of origin of the participant. Participants in low/middle income countries are taken as the reference category; estimates for high income countries are deviations from the estimate for low/middle income countries. The interaction term indicates the significance of the interaction between age of onset/severity and country income group.

* p < 0.005 ** p < 0.001

Supplemental table 18: Associations between internalizing classes (posterior probabilities of class membership) and 12-month suicidal ideation (outcome), by country income group.

| **Class** | | **Low/middle income countries**  **(reference group)** | | **High income countries**  **(vs. low/middle)** | | **Test for interaction between income group and posterior probabilities** | |
| --- | --- | --- | --- | --- | --- | --- | --- |
|  |  | *% (SE)* | *OR (95% CI)* | *% (SE)* | *OR (95% CI)* | *F(1,1157)* | *P-value* |
| **Healthy** |  | 0.8 (0.1) | 1.0 | 0.7 (0.1) |  | - | - |
| **Mild** | **Childhood onset** | 4.4 (1.7) | 5.5 (2.2-13.9)** | 2.7 (0.6) | 0.7 (0.2-2.0) | 0.4 | 0.521 |
|  | **Puberty onset** | 4.3 (1.1) | 5.7 (3.0-10.7)** | 3.3 (0.6) | 0.9 (0.4-1.9) | 0.1 | 0.700 |
|  | **Early-adult onset** | 2.3 (0.9) | 3.0 (1.3-6.9) | 3.8 (0.8) | 1.9 (0.7-4.9) | 1.6 | 0.206 |
|  | **Middle-adult onset** | 4.1 (1.1) | 5.4 (2.9-10.3)** | 2.6 (1.0) | 0.7 (0.3-2.0) | 0.4 | 0.504 |
| **Moderate** | **Childhood onset** | 6.3 (1.8) | 7.2 (3.4-15.3)** | 6.7 (1.0) | 1.5 (0.6-3.5) | 0.8 | 0.383 |
|  | **Puberty onset** | 5.7 (1.9) | 7.8 (3.5-17.0)** | 3.7 (1.0) | 0.7 (0.2-1.8) | 0.7 | 0.402 |
| **Severe** | **Childhood onset** | 18.6 (5.2) | 27.2 (12.9-57.4)** | 12.6 (1.9) | 0.7 (0.3-1.8) | 0.5 | 0.498 |

Table notes: Percentages are weighted percentages derived from cross-tabulation. Tests were based on logistic regression with 12-month suicidal ideation as an outcome and the posterior probabilities of class membership as predictors, controlling for country of origin of respondents. Participants in low/middle income countries are taken as the reference category; estimates for high income countries are deviations from the estimate for low/middle income countries. The interaction term indicates the significance of the interaction between each probability and country income group. Note: suicidal plan and suicide attempt were not tested, due to low prevalence.

N = unweighted sample size per class (when respondents are assigned to their most likely class). * p<0.005 ** p<0.001

Supplemental table 19: Logistic regression analysis of the association between age of onset and severity of each class (predictors) and 12-month suicidal ideation (outcome), by country income group

| **Variable** | **Level** | **Low/middle income countries**  **(reference group)** | **High income countries**  **(vs. low/middle)** | **Test for interaction between income group and posterior probabilities** | |
| --- | --- | --- | --- | --- | --- |
|  |  | **OR (95% CI)** | **OR (95% CI)** | **F(2,1156)** | **P-value** |
| **Age of onset** | Healthy | 0.2 (0.1-0.4)** | 1.2 (0.5-2.9) | 0.4 | 0.650 |
|  | Childhood | 1.0 | 1.0 |  |  |
|  | Puberty | 1.0 (0.5-2.1) | 0.8 (0.4-1.9) |  |  |
|  | Adult | 0.8 (0.3-1.8) | 1.2 (0.5-3.3) |  |  |
| **Severity** | Mild | 1.0 | 1.0 | 0.4 | 0.655 |
|  | Moderate | 1.4 (0.7-2.7) | 1.3 (0.6-3.0) |  |  |
|  | Severe | 4.7 (1.8-12.4)* | 0.9 (0.3-2.6) |  |  |

Table notes: Age of onset and severity were entered into the model simultaneously. All analyses controlled for country of origin of the participant. Participants in low/middle income countries are taken as the reference category; estimates for high income countries are deviations from the estimate for low/middle income countries. The interaction term indicates the significance of the interaction between age of onset/severity and country income group. Note: suicidal plan and suicide attempt were not tested, due to low prevalence.

* p < 0.005 ** p < 0.001

Supplemental table 20: Associations between internalizing classes (posterior probabilities of class membership) and 12-month treatment in any sector (outcome), by country income group.

| **Class** | | **Low/middle income countries**  **(reference group)** | | **High income countries**  **(vs. low/middle)** | | **Test for interaction between income group and posterior probabilities** | |
| --- | --- | --- | --- | --- | --- | --- | --- |
|  |  | *% (SE)* | *OR (95% CI)* | *% (SE)* | *OR (95% CI)* | *F(1,1157)* | *P-value* |
| **Healthy** |  | 3.4 (0.3) | 1.0 | 7.3 (0.3) |  | - | - |
| **Mild** | **Childhood onset** | 12.5 (2.0) | 3.4 (2.3-5.2)** | 21.2 (1.6) | 1.0 (0.6-1.6) | 0.0 | 0.930 |
|  | **Puberty onset** | 14.7 (2.2) | 4.6 (3.1-6.8)** | 21.7 (1.5) | 0.7 (0.5-1.2) | 1.7 | 0.191 |
|  | **Early-adult onset** | 15.1 (2.5) | 5.2 (3.3-8.1)** | 30.6 (2.0) | 1.1 (0.7-1.7) | 0.1 | 0.796 |
|  | **Middle-adult onset** | 21.0 (3.7) | 7.1 (4.4-11.5)** | 26.0 (1.9) | 0.6 (0.4-1.0) | 3.4 | 0.065 |
| **Moderate** | **Childhood onset** | 27.2 (3.8) | 9.8 (6.3-15.0)** | 39.5 (2.2) | 0.9 (0.5-1.4) | 0.3 | 0.568 |
|  | **Puberty onset** | 25.3 (4.7) | 8.1 (4.8-13.5)** | 42.3 (2.6) | 1.1 (0.6-2.0) | 0.1 | 0.713 |
| **Severe** | **Childhood onset** | 20.6 (4.3) | 6.7 (3.9-11.7)** | 55.1 (2.7) | 2.2 (1.2-4.0) | 6.7 | 0.010 |

Table notes: Percentages are weighted percentages derived from cross-tabulation. Tests were based on logistic regression with 12-month treatment in any sector as an outcome and the posterior probabilities of class membership as predictors, controlling for country of origin of respondents. Participants in low/middle income countries are taken as the reference category; estimates for high income countries are deviations from the estimate for low/middle income countries. The interaction term indicates the significance of the interaction between each probability and country income group. Note: only treatment in any sector and treatment in specialist mental health care were tested, due to low prevalence and/or model convergence problems with other sectors.

N = unweighted sample size per class (when respondents are assigned to their most likely class). * p<0.005 ** p<0.001

Supplemental table 21: Logistic regression analysis of the association between age of onset and severity of each class (predictors) and 12-month treatment in any sector (outcome), by country income group

| **Variable** | **Level** | **Low/middle income countries**  **(reference group)** | **High income countries**  **(vs. low/middle)** | **Test for interaction between income group and posterior probabilities** | |
| --- | --- | --- | --- | --- | --- |
|  |  | **OR (95% CI)** | **OR (95% CI)** | **F(2,1156)** | **P-value** |
| **Age of onset** | Healthy | 0.3 (0.2-0.4)** | 1.1 (0.7-1.7) | 0.1 | 0.873 |
|  | Childhood | 1.0 | 1.0 |  |  |
|  | Puberty | 1.1 (0.8-1.7) | 0.9 (0.6-1.4) |  |  |
|  | Adult | 1.6 (1.0-2.6) | 0.9 (0.5-1.5) |  |  |
| **Severity** | Mild | 1.0 | 1.0 | 3.6 | 0.027 |
|  | Moderate | 2.3 (1.6-3.4)** | 1.1 (0.7-1.7) |  |  |
|  | Severe | 1.7 (0.9-3.3) | 2.6 (1.3-5.3) |  |  |

Table notes: Age of onset and severity were entered into the model simultaneously. All analyses controlled for country of origin of the participant. Participants in low/middle income countries are taken as the reference category; estimates for high income countries are deviations from the estimate for low/middle income countries. The interaction term indicates the significance of the interaction between age of onset/severity and country income group. Note: only treatment in any sector and treatment in specialist mental health care were tested, due to low prevalence and/or model convergence problems with other sectors.

* p < 0.005 ** p < 0.001

Supplemental table 22: Associations between internalizing classes (posterior probabilities of class membership) and 12-month treatment in specialist mental health care (outcome), by country income group.

| **Class** | | **Low/middle income countries**  **(reference group)** | | **High income countries**  **(vs. low/middle)** | | **Test for interaction between income group and posterior probabilities** | |
| --- | --- | --- | --- | --- | --- | --- | --- |
|  |  | *% (SE)* | *OR (95% CI)* | *% (SE)* | *OR (95% CI)* | *F(1,1157)* | *P-value* |
| **Healthy** |  | 1.2 (0.2) | 1.0 | 2.8 (0.2) |  | - | - |
| **Mild** | **Childhood onset** | 4.7 (1.3) | 3.1 (1.5-6.4)* | 8.3 (1.0) | 1.0 (0.5-2.3) | 0.0 | 0.910 |
|  | **Puberty onset** | 7.5 (2.0) | 6.0 (3.0-11.8)** | 8.6 (0.9) | 0.5 (0.3-1.1) | 2.7 | 0.100 |
|  | **Early-adult onset** | 6.8 (1.4) | 6.5 (3.9-11.0)** | 12.9 (1.3) | 0.8 (0.5-1.5) | 0.4 | 0.523 |
|  | **Middle-adult onset** | 8.2 (1.8) | 6.6 (3.6-12.1)** | 9.4 (1.2) | 0.5 (0.3-1.1) | 3.1 | 0.079 |
| **Moderate** | **Childhood onset** | 14.6 (2.7) | 10.7 (6.0-19.2)** | 15.2 (1.6) | 0.6 (0.3-1.2) | 1.8 | 0.176 |
|  | **Puberty onset** | 12.0 (3.3) | 8.8 (4.8-16.3)** | 15.7 (2.1) | 0.8 (0.4-1.5) | 0.6 | 0.438 |
| **Severe** | **Childhood onset** | 12.4 (3.2) | 8.4 (4.4-16.1)** | 24.2 (2.2) | 1.4 (0.7-2.9) | 0.8 | 0.366 |

Table notes: Percentages are weighted percentages derived from cross-tabulation. Tests were based on logistic regression with 12-month specialist mental health care as an outcome and the posterior probabilities of class membership as predictors, controlling for country of origin of respondents. Participants in low/middle income countries are taken as the reference category; estimates for high income countries are deviations from the estimate for low/middle income countries. The interaction term indicates the significance of the interaction between each probability and country income group. Note: only treatment in any sector and treatment in specialist mental health care were tested, due to low prevalence and/or model convergence problems with other sectors.

N = unweighted sample size per class (when respondents are assigned to their most likely class). * p<0.005 ** p<0.001

Supplemental table 23: Logistic regression analysis of the association between age of onset and severity of each class (predictors) and 12-month treatment in specialist mental health care (outcome), by country income group

| **Variable** | **Level** | **Low/middle income countries**  **(reference group)** | **High income countries**  **(vs. low/middle)** | **Test for interaction between income group and posterior probabilities** | |
| --- | --- | --- | --- | --- | --- |
|  |  | **OR (95% CI)** | **OR (95% CI)** | **F(2,1156)** | **P-value** |
| **Age of onset** | Healthy | 0.3 (0.1-0.5)** | 1.2 (0.6-2.3) | 0.5 | 0.582 |
|  | Childhood | 1.0 | 1.0 |  |  |
|  | Puberty | 1.3 (0.8-2.4) | 0.7 (0.4-1.4) |  |  |
|  | Adult | 1.7 (0.9-3.2) | 0.8 (0.4-1.5) |  |  |
| **Severity** | Mild | 1.0 | 1.0 | 1.3 | 0.269 |
|  | Moderate | 2.4 (1.4-4.1)* | 0.9 (0.5-1.6) |  |  |
|  | Severe | 2.1 (0.9-4.9) | 1.7 (0.7-4.2) |  |  |

Table notes: Age of onset and severity were entered into the model simultaneously. All analyses controlled for country of origin of the participant. Participants in low/middle income countries are taken as the reference category; estimates for high income countries are deviations from the estimate for low/middle income countries. The interaction term indicates the significance of the interaction between age of onset/severity and country income group. Note: only treatment in any sector and treatment in specialist mental health care were tested, due to low prevalence and/or model convergence problems with other sectors.

* p < 0.005 ** p < 0.001
